# Spectroscopic and elemental evaluation of Pb(II), Cu(II), and Hg(II) binding by *salvia officinalis* L. leaf extract

**DOI:** 10.1186/s13065-026-01878-y

**Published:** 2026-07-11

**Authors:** Huri Tasci, Melek Gul, Ebru Batı Ay, Beril Kocaman

**Affiliations:** 1 Pistachio Research Institute, Republic of Türkiye Ministry of Agriculture and Forestry, Şahinbey, 27070 Gaziantep, Türkiye; 2 Department of Chemistry, Faculty of Art and Sciences, Amasya University, Amasya, Türkiye; 3 Mathematics and Science Education Department, Faculty of Education, Amasya University, Amasya, 05100 Türkiye; 4 Department of Plant and Animal Production, Suluova Vocational School, Amasya University, Suluova, Amasya, Türkiye

**Keywords:** Chelation, Heavy metals, Metal complexation, Phytoremediation, Secondary metabolites

## Abstract

Heavy metal contamination remains a major environmental concern because toxic ions such as Pb(II), Cu(II), and Hg(II) persist in ecosystems and pose serious risks to the environment and human health. Plant-derived extracts rich in phenolic acids, flavonoids, and other heteroatom-containing phytochemicals offer a promising natural matrix for metal binding through hydroxyl, carbonyl, and C–O-containing functional groups. This study presents a comparative matrix-level evaluation of *interactions of Pb(II)*,* Cu(II)*,* and Hg(II) with* *Salvia officinalis *L. leaf extract by integrating UV–Vis spectroscopic stoichiometry, apparent binding analysis, pH- and temperature-dependent spectral responses, EDTA-assisted reversibility, FTIR functional group assignments, and ICP-OES-based elemental validation. UV–Vis data were evaluated using Job’s plot analysis, while apparent binding parameters were estimated using the extended Benesi–Hildebrand and Scatchard models, based on the observed stoichiometric behaviour. Pb(II) and Hg(II) exhibited apparent M₂L-type interaction patterns, whereas Cu(II) showed a 1:1 binding mode. Among the tested ions, Hg(II) produced the strongest spectroscopic binding response, followed by Pb(II) and Cu(II). The metal–extract interactions were strongly affected by pH and temperature, with more pronounced spectral responses under mildly alkaline conditions and at temperatures above 45 °C. EDTA addition indicated that the binding process was at least partially reversible, suggesting the potential regeneration of the extract-based metal-binding system. ICP-OES analysis supported the incorporation of metals into the extract-derived complexes, whereas changes in FTIR spectra indicated the involvement of hydroxyl, carbonyl, and C–O groups in metal coordination. Overall, the findings demonstrate the metal-dependent binding behavior of *S. officinalis* leaf extract and provide a useful spectroscopic and elemental basis for further studies on plant-derived metal-binding systems.

## Introduction

 Heavy metal contamination of soil and aquatic environments has become one of the most pressing global environmental challenges due to rapid industrialisation, mining activities, agricultural inputs, and urban development. Toxic metals such as mercury(Hg), lead(Pb), cadmium(Cd), chromium(Cr), and copper(Cu)are non-biodegradable and tend to accumulate in ecosystems, posing serious risks to environmental sustainability and human health, even at trace levels [1]. Conventional remediation technologies, including chemical precipitation, ion exchange, and membrane filtration, are often limited by high operational costs, secondary waste generation, and low selectivity, which have driven increasing interest in alternative, eco-friendly remediation strategies.

In this context, biosorption has emerged as a promising and sustainable approach for the removal of heavy metals. Biosorbents are natural or chemically modified biological materials that passively adsorb and accumulate metal ions from aqueous environments via interactions between the ions and functional groups on the biosorbent surface. A wide range of biological materials, including algae, fungi, bacteria, and plant tissues such as leaves, stems, and fruits, has been investigated as effective biosorbents [2, 3]. Plant-based biosorbents are particularly attractive compared with conventional adsorbents such as activated carbon, synthetic ion-exchange resins, mineral materials (e.g., zeolite and bentonite), and microbial biosorbents because of their lower cost, renewability, and a high abundance of naturally occurring metal-binding functional groups [2, 4, 5].

Medicinal and aromatic plants(MAPs)have gained special attention in biosorption studies owing to their rich phytochemical compositions, which include phenolic acids, flavonoids, terpenoids, and other secondary metabolites with strong metal-chelating properties [6, 7]. Moreover, exposure to abiotic stressors, such as heavy metal contamination, has been reported to stimulate the biosynthesis of secondary metabolites, potentially enhancing the medicinal value of these plants while contributing to environmental detoxification [8]. This dual benefit makes MAPs promising candidates for integrated environmental and biotechnological applications.

Among MAPs, *S. officinalis L*. (sage), a member of the Lamiaceae family, is well known for its culinary, medicinal, antioxidant, antimicrobial, and anti-inflammatory properties. Its leaves are rich in phenolic compounds such as rosmarinic acid, caffeic acid, and various flavonoids, which have a strong affinity for metal ions [9, 10]. Recent experimental studies have demonstrated that S. o*fficinalis extr*acts exhibit considerable biosorption capacity toward toxic metals, including Pb, Cd, and Cr, highlighting their potential as low-cost and sustainable biosorbents [11].

Spectroscopic techniques, particularly UV–Vis spectroscopy, have been widely employed to investigate metal–extract interactions by monitoring changes in absorbance upon metal binding. Shifts in characteristic absorption bands and the emergence of new spectral features provide clear *evidence that heavy metal ions form stable metal–ligand complexes with phytochemical constituents of S. officinalis* [11]. The biosorption mechanism is mainly attributed to functional groups such as phenolic hydroxyls, flavonoids, and terpenoids that effectively coordinate metal ions and enhance adsorption efficiency.

Despite these advances, detailed molecular-level insights into the coordination pathways and metal selectivity of *S. officinalis* phytochemicals remain limited. Most existing studies focus on adsorption capacity and removal efficiency, while the mechanistic aspects of metal–ligand interactions are often insufficiently addressed [12]. A deeper understanding of these interactions is essential for evaluating the selectivity and potential application of plant-derived compounds as targeted chelating agents in environmental remediation.

Plant-derived extracts have increasingly been used in green chemistry-based environmental applications because their phenolic compounds, flavonoids, alkaloids, organic acids, and other oxygen-/nitrogen-containing biomolecules can act as reducing, stabilizing, capping, or metal-interacting [13–15]. For example, *Camelia sinensis* leaf extract was reported to mediate the synthesis of Cu nanoparticles, where plant biomolecules were responsible for reduction and stabilization, and FTIR bands associated with O–H, aromatic phenolic, and C–O groups were linked to these interactions [13]. Similarly, *Ficus carica* peel extract was used for the green synthesis of Fe nanoparticles, and the authors associated the process with hydroxyl, carbonyl, C = C, and C–O vibrations of phytochemical constituents that may contribute to capping and stabilization [15]. In another study, Fe nanoparticles biosynthesized using *Actinidia chinensis* fruit extract showed that polyphenols, flavonoids, and phenolic acids can participate in metal reduction and stabilization through hydroxyl-rich structures, while FTIR and surface analyses supported the involvement of plant-derived biomolecules in nanoparticle formation and environmental dye degradation applications [13]. These reports support the broader concept that plant extracts can serve as chemically active matrices in metal-related environmental systems, not only through nanoparticle formation but also through metal coordination, stabilization, and surface- and solution-phase interactions. Therefore, the present study *of the Salvia* *officinalis* leaf extract aligns with this green chemistry framework and further contributes by examining comparative solution-phase interactions of Pb(II), Cu(II), and Hg(II)using UV–Vis, FTIR, and ICP-OES.

In this study, the interaction between *S. officinalis* leaf extract and selected transition and heavy metal ions—Hg(II), Cu(II), and Pb(II)—was systematically investigated using UV–Vis spectroscopy. The aim was to elucidate the complexation behaviour, binding affinity, and stoichiometry of metal–ligand interactions. Metal selectivity and binding efficiency were assessed through pH- and temperature-dependent studies, complemented by investigations of the reversibility of metal–ligand interactions. pH is a key parameter influencing metal selectivity and binding efficiency, as it modulates metal interactions and the availability of ligand binding sites [16]. Variations in pH can therefore significantly alter the strength and selectivity of metal–ligand interactions in complex environmental systems, such as plant extracts. The reversibility of metal complexation in the presence of chelating agents such as ethylenediaminetetraacetic acid(EDTA)contributes to environmental remediation and phytoremediation, as it governs metal mobility, desorption control, and phytoextraction efficiency [17, 18]. EDTA is widely employed due to its strong affinity for divalent and trivalent metal ions [19]. By clarifying the selective chelation properties of *S. officinalis*, this work seeks to contribute to the development of eco-friendly, plant-based materials for heavy metal remediation and to expand the functional applications of medicinal plants beyond their traditional uses. In this study, the mechanistic behavior of crude *S. officinalis* leaf extract toward selected metal ions was evaluated through (i) metal selectivity experiments, (ii)Job’s plot analysis, (iii)pH-dependent complexation studies, (iv)temperature-dependent UV–Vis measurements, (v)time-dependent interaction studies, (vi)EDTA-assisted reversibility tests, and(vii)comparison with quercetin, a structurally defined positive control. The results were supported by UV–Vis, FTIR, and ICP-OES analyses, which were used to clarify the possible role of phytochemical donor groups in metal coordination.

## Materials and methods

### Material

The study was conducted from 2024 to 2025 in the plastic greenhouse of the Department of Plant and Animal Production at Amasya University. *S. officinalis L. was* used as the plant material in the experiment. Sage seedlings were obtained from a commercial nursery located in Yalova, Türkiye. Plants were grown in 3-L pots filled with a peat–perlite mixture (1:1, v/v), and the seedlings were transplanted in April. From planting onward, routine cultural practices were applied. During the cultivation period, weeds growing in the pots were manually removed. Leaf samples were collected at the end of the vegetation period. The leaves were washed in the laboratory and then dried in paper bags in a drying oven at 40 °C.

### Method

#### Plant extraction method

The plant samples, dried under appropriate conditions, were ground using a manual grinder. During the extraction stage, 10 g of the plant sample were weighed on an analytical balance and transferred to sealed glass jars; 200 mL of methanol was then added, and the mixtures were shaken until a homogeneous suspension was obtained. Subsequently, the samples were subjected to ultrasonic-assisted extraction for two hours. The ultrasonic method was selected because of its rapid, highly efficient performance at low temperatures and its ability to preserve the structural integrity of extracts and to prevent degradation of bioactive compounds. After extraction, the supernatant was filtered and the solvent removed by rotary evaporation to yield a concentrated extract.

#### Total flavonoid content

The total flavonoid content was determined according to the method described by Park et al. [20] using quercetin as the reference standard. For the analysis, 1 mL of each plant extract was transferred into test tubes. Subsequently, 2 mL of distilled water, 0.15 mL of 0.5 M NaNO₂ solution, and 0.15 mL of 0.3 M AlCl₃ solution were added. After the mixture was allowed to stand for 5 min, 1 mL of NaOH solution was added. The absorbance of the resulting colored complex was measured at 510 nm using a spectrophotometer. The recorded absorbance values were compared with a quercetin calibration curve, and the total flavonoid content was expressed as milligrams of quercetin equivalents per gram of dried plant material (mg QE/g).

#### Total phenolic content

The total phenolic content was determined using the Folin–Ciocalteu assay, originally described by Otto Folin and Vintilă Ciocâlteu [21]. For this purpose, 0.1 mL of each extract solution (1 mg/mL) was transferred into test tubes, and 4.5 mL of distilled water was added. Subsequently, 0.1 mL of Folin–Ciocalteu reagent and 0.3 mL of 2% sodium carbonate solution were added, and the mixture was thoroughly homogenized. The samples were kept in the dark for two hours to allow the reaction to proceed to completion. Absorbance measurements were then carried out at 760 nm against distilled water, following the procedure reported by Slinkard and Singleton [22]. Total phenolic content was calculated using a gallic acid calibration curve and expressed as milligrams of gallic acid equivalents per gram of dried sample(mg GAE/g).

#### Preparation of metal–extract solutions

The S. *officinalis leaf* extract was prepared in dimethyl sulfoxide (DMSO) at 0.4 mg/mL and used as the crude extract matrix for metal-binding experiments. Fresh Pb(II), Cu(II), and Hg(II) solutions were prepared at 1 × 10⁻⁵ M in DMSO. For Job’s continuous-variation experiments, metal ion and extract solutions were mixed at different mole fractions while keeping the total volume constant. Because the plant extract contains a chemically complex mixture of phytochemicals rather than a structurally defined single ligand, the resulting stoichiometric ratios were interpreted as apparent metal–extract interaction patterns. Absorbance values were recorded after 10 min of equilibration [23].

#### UV–Vis spectroscopic measurements

UV–Vis spectra were collected in the range of 200–600 nm. The free crude extract matrix exhibited two major absorption bands at 247 nm and 411 nm [24, 25].

#### Evaluation of relative UV–Vis spectral response

The relative UV–Vis spectral response of the *S. officinalis* extract to *Pb(II)*, Cu(II), and Hg(II) was evaluated by considering both changes in absorbance intensity and shifts in wavelength following metal-ion addition. Since an independent adsorption selectivity coefficient was not calculated, the term selectivity was replaced with relative UV–Vis spectral response. The wavelength shift was calculated as:

$$\:{\Delta\:}{\lambda\:}_{\mathrm{m}\mathrm{a}\mathrm{x}}={\lambda\:}_{\mathrm{m}\mathrm{a}\mathrm{x},\mathrm{metal\--extract}}-{\lambda\:}_{\mathrm{m}\mathrm{a}\mathrm{x},\mathrm{extract}}$$

and the absorbance change was calculated as:

$$\:{\Delta\:}A={A}_{\mathrm{metal\--extract}}-{A}_{\mathrm{extract}}$$

$\:{\lambda\:}_{\mathrm{m}\mathrm{a}\mathrm{x},\mathrm{metal\--extract}}$The symbols $\:{A}_{\mathrm{metal\--extract}}$represent the maximum absorption wavelength and absorbance of the metal–extract system, while $\:{\lambda\:}_{\mathrm{m}\mathrm{a}\mathrm{x},\mathrm{extract}}$other symbols $\:{A}_{\mathrm{extract}}$represent those of the free extract under the same experimental conditions. Positive $\:{\Delta\:}{\lambda\:}_{\mathrm{m}\mathrm{a}\mathrm{x}}$values indicate bathochromic shifts, whereas negative values indicate hypsochromic shifts. These parameters were used only as comparative spectroscopic descriptors of the crude extract matrix with respect to Pb(II), Cu(II), and Hg(II), and not as true adsorption selectivity coefficients(Table 1).

**Table 1:** Relative UV–Vis spectral response of *S. officinalis* extract after addition of Pb(II), Cu(II), and Hg(II)

Sample	Main λmax (nm)	A at main λmax	Δλmax vs. extract (nm)	A₃₁₁	ΔA₃₁₁	A₄₁₁	ΔA₄₁₁	A₆₄₄	ΔA₆₄₄
Extract	257	1.8050	–	1.4923	–	0.1991	–	0.0116	–
Hg(II)–Extract	249	2.5719	−8	1.1393	−0.3530	0.2349	+ 0.0358	0.1215	+ 0.1099
Cu(II)–Extract	258	1.6323	+ 1	1.1660	−0.3263	0.3688	+ 0.1697	0.1905	+ 0.1789
Pb(II)–Extract	242	2.0327	−15	1.0657	−0.4266	0.2361	+ 0.0370	0.0827	+ 0.0711

#### Job’s method of continuous variation

Absorbance changes were monitored at the shifted wavelength, and the Job’s plot was constructed using Eq. 1:

1 $$\:{X}_{M}=\frac{\left[M\right]}{\:\left[M\right]+\left[L\right]}$$

The maximum absorbance change observed in the Job’s plot was used to estimate the apparent metal-to-extract interaction ratio. Since the extract is a chemically complex matrix rather than a purified ligand, these ratios were interpreted only as apparent stoichiometric trends. In this context, Pb(II) and Hg(II) showed maximum responses at metal mole fractions close to 0.67, suggesting a higher metal contribution in the interaction pattern, whereas Cu(II) showed a maximum response at approximately 0.50, indicating a more balanced metal–extract interaction profile.

#### Comparison with quercetin as positive control ligands

To provide a structurally defined comparison for the metal-binding behavior of the crude plant extract, quercetin was used as a positive control ligand. Quercetin stock solution (7,0 × 10⁻⁵ M) was prepared in DMSO for comparison with the plant extract study. Metal ion solutions of Cu(II), Pb(II), and Hg(II) were prepared separately in distilled water. For the UV–Vis binding comparison, quercetin solution was mixed with increasing equivalents of each metal ion under identical solvent conditions, keeping the final solvent ratio constant across all measurements. The spectra of free quercetin and quercetin–metal mixtures were recorded by UV–Vis spectroscopy over the 200–800 nm range. For the estimation of apparent metal-to-ligand stoichiometry, Job’s method of continuous variation was applied similarly to the plant matrix.

#### Determination of reversibility

The reversibility of the plant extract–metal (Pb, Hg, and Cu²⁺) complexes was evaluated using EDTA as a competing chelating agent under controlled experimental conditions. The UV–Vis spectra of the free plant extract and of the systems containing the selected metal ions were initially recorded and characterized. EDTA concentrations ranging from 0.5 to 10 equivalents relative to the metal were then added to assess reversibility and to monitor changes in the UV–Vis absorption spectra [26].

#### Determination of the pH effect on the metal absorption

To evaluate the effect of pH on metal–extract interactions, UV–Vis spectroscopic experiments were carried out using *S. officinalis *extract solutions (200 ppm), in the presence and absence of Cu(II), Pb(II), and Hg(II) ions (each at 2 × 10⁻⁵ M). The pH was adjusted over the range of 2–12 using appropriate sodium carbonate buffer solutions and monitored with a calibrated pH meter. To minimize variations in ionic strength during pH adjustment, NaCl was used as a background electrolyte, and the final NaCl concentration was maintained at 0.1 M in all sample solutions. The final volume of each sample was adjusted to 2 mL with DMSO after the addition of the extract, metal ion solution, buffer, and NaCl. The solutions were equilibrated under the same experimental conditions, and their UV–Vis spectra were then recorded [27].

#### Determination of binding constants

- Extended Benesi–Hildebrand Method for ML₂ complexes

The apparent binding response of the Pb(II)–extract and Hg(II)–extract systems was evaluated using an extended Benesi–Hildebrand approach. Since *S. officinalis leaf* extract is a chemically complex, crude matrix rather than a purified ligand with a defined molar concentration, the calculated values were interpreted as apparent binding parameters rather than as true thermodynamic association constants. In this analysis, A₀ represents the absorbance of the free extract matrix, A is the absorbance after metal ion addition, ΔAmax is the theoretical maximum absorbance change, and [M] is the free metal ion concentration. The linear plot of 1/(A₀ − A) versus 1/[M]² was used to compare the apparent binding responses of the metal–extract systems. The extended Benesi–Hildebrand equation (Eq. 2) was expressed as follows:

2 $$\:\frac{1}{{A}_{0}-A}+\frac{1}{\varDelta\:{\:A}_{max}}+\frac{1}{{K}_{app}\:\varDelta\:{\:A}_{max}}.\frac{1}{{\left[M\right]}^{2}}$$

where $\:{K}_{app}$represents the apparent binding parameter obtained under the applied experimental conditions.

(b) Scatchard Analysis

The Cu(II)–extract interaction was evaluated by Scatchard analysis because the Job’s plot suggested an apparently balanced metal–extract interaction pattern. The Scatchard model was used only as a comparative approach to estimate the apparent binding response and the relative availability of binding sites within the crude extract matrix [28, 29]. In this equation, r represents the fraction of bound metal ions; $\:{K}_{app}$[M] represents the concentration of free metal ions; the apparent binding parameter is indicated in the equation; and n represents the apparent number of binding sites within the extract matrix.

The Scatchard Eq. 3 was used:

3 $$\:\frac{r}{\left[M\right]}={K}_{app}\left(n-r\right)\:\:\:\:\:$$

#### Limit of detection (LOD) and limit of quantification (LOQ)

Limit of detection LOD and limit of quantification LOQ were calculated using the standard IUPAC equations, where σ is the standard deviation of three replicate blank measurements, and slope refers to the sensitivity of the method, obtained from the calibration curve (Abs vs. [M])

LOD and LOQ were calculated from the calibration plot using Eq. 4:

4 $$\:LOD=\frac{3.3\sigma\:}{slope}\:\:\:LOQ=\frac{10\sigma\:}{slope}\:\:\:\:$$

The absorbance-based limits of detection (LOD) and quantification (LOQ) were calculated for each metal system, yielding LOD/LOQ values, expressed in absorbance (A), of 2.001/2.059 A for Pb(II), 2.241/2.357 A for Hg(II), and 0.277/0.315 A for Cu(II), respectively.

#### Preparation of metal–extract complexes and ICP-OES analysis

The plant extract was prepared and subsequently used for metal-complexation studies. For Cu–extract complex formation, 155 mg of extract and 13.3 mg of Cu(NO₃)₂ were reacted; for Pb–extract complex formation, 180 mg of extract and 19 mg of Pb(NO₃)₂ were reacted. In both cases, the metal salt was dissolved in an aqueous medium and added to the ethanolic extract solution under continuous stirring. The mixtures were allowed to react until visible precipitation occurred, after which the resulting solid complexes were separated by filtration, washed with distilled water to remove unbound metal ions, and dried at room temperature. The final masses of the isolated complexes were 86 mg for the Cu–extract complex and 92 mg for the Pb–extract complex.

Prior to ICP-OES analysis (Perkin-Elmer/ICP-OES Optima 8300), native plant extract samples, metal-spiked extract solutions (external standard added at 10 ppm), and isolated metal–extract complexes were subjected to wet digestion. All samples were digested with 2 mL of concentrated HNO₃ and 1 mL of H₂O₂ under controlled heating at 120 °C until complete mineralisation was achieved (35–45 min). After digestion, the solutions were diluted to the appropriate volume with ultrapure water and analysed by ICP-OES. Non-spiked digested samples were used to determine the intrinsic metal content. In contrast, spiked samples were prepared by externally adding 10 ppm metal standards to evaluate matrix effects and analytical accuracy. All ICP-OES measurements were performed in triplicate.

### Statistical analysis

A one-way analysis of variance (ANOVA) was used to evaluate the effect of pH on the UV–Vis absorbance values of the free extract and metal–extract systems. Measurements were evaluated as mean ± standard deviation (SD). When significant differences were detected, Tukey’s honestly significant difference (HSD) post hoc test was applied to conduct pairwise comparisons between pH levels. Statistical significance was accepted at *p* < 0.05 (Table 2). Different superscript letters within the same row indicate statistically significant differences among pH levels at *p* < 0.05, while values sharing at least one common letter are not significantly different.

**Table 2:** Mean ± SD absorbance values of *Salvia officinalis* leaf extract and metal–extract systems at different pH values

Sample	pH 2	pH 4	pH 6	pH 8	pH 10	pH 12
Extract	0.525 ± 0.018^c^	0.531 ± 0.007^c^	1.565 ± 0.154^a^	0.651 ± 0.143^bc^	0.769 ± 0.203^bc^	0.863 ± 0.223^b^
Pb(II) extract	1.635 ± 0.049^d^	2.167 ± 0.060^b^	0.967 ± 0.057^e^	2.452 ± 0.110^a^	1.898 ± 0.047^c^	1.540 ± 0.175^d^
Cu(II) extract	1.676 ± 0.111^d^	2.144 ± 0.077b	1.012 ± 0.085^f^	2.378 ± 0.148^a^	1.936 ± 0.084^c^	1.487 ± 0.071^e^
Hg(II) extract	0.572 ± 0.074^d^	1.333 ± 0.219c	0.808 ± 0.106^d^	2.442 ± 0.388^a^	1.681 ± 0.240^bc^	2.031 ± 0.298^ab^

Different superscript letters within the same row indicate significant differences according to Tukey’s HSD post hoc test (*p* < 0.05)

## Results discussion

**Fig. 1:**
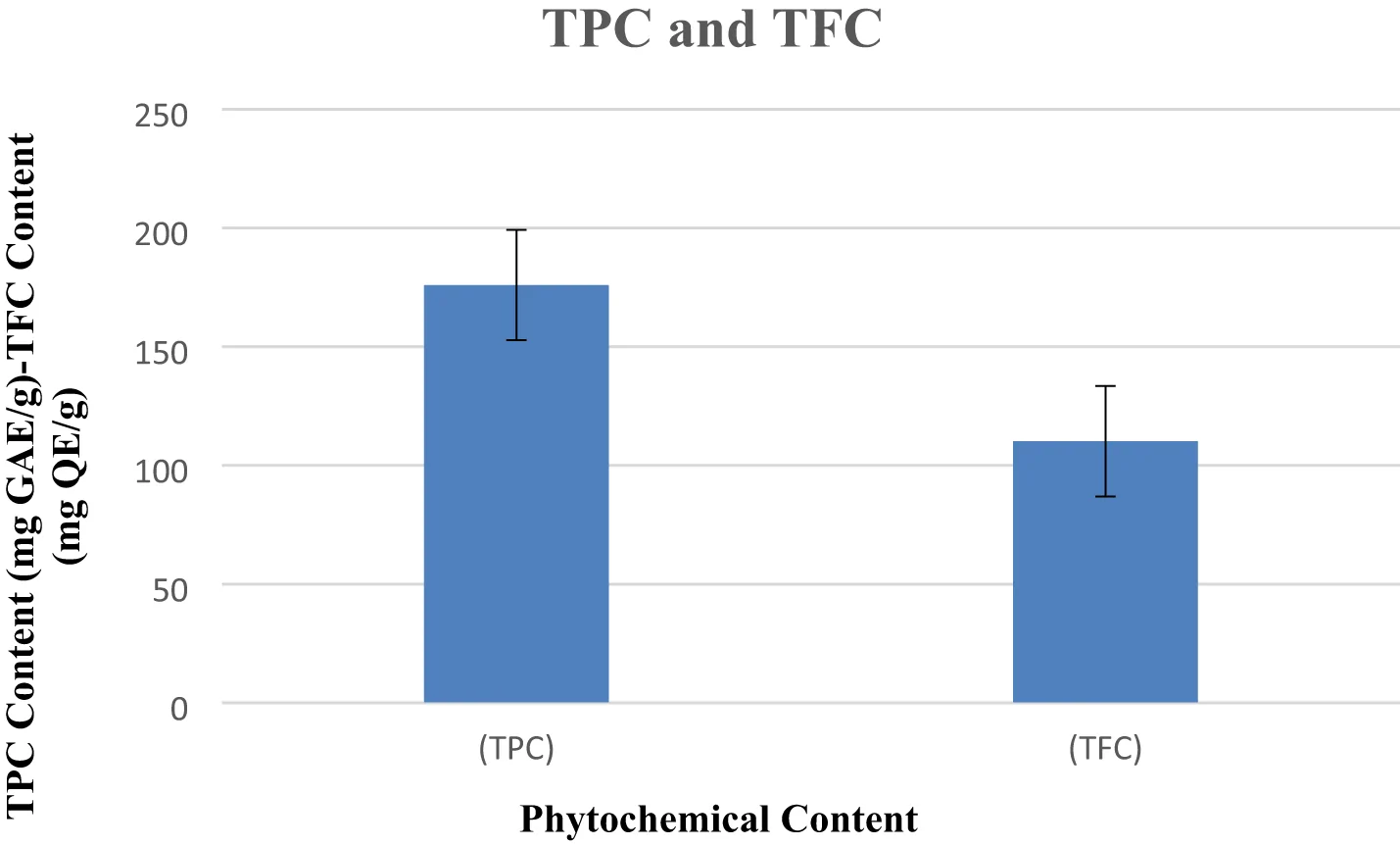
Total phenolic and total flavonoid content of *S. officinalis* leaf extract

*S. officinalis* leaves possess abundant functional groups (–OH, –COOH) owing to their high total phenolic and flavonoid content (e.g., rosmarinic acid, carnosic acid, and luteolin derivatives). These functional groups facilitate binding through complexation and electrostatic interactions with metal ions and organic pollutants; therefore, the plant’s phenolic-rich biomass has been reported in various studies as a promising, low-cost biosorbent material [30–32]. In our study, the *total phenolic content* (TPC) of S. *officinalis* was 1759.67 mg GAE/g, while the total flavonoid content (TFC) was 1101.89 mg QE/g (Fig. 1). These values indicate that the plant is particularly rich in phenolic compounds. Phenolic groups can form coordination bonds with transition metal ions such as Pb²⁺, Cu²⁺, and Ni²⁺. For instance, Aityoub et al. [18] emphasized the importance of physicochemical factors and organic functional groups in the removal of Pb and Cu from soil environments. Similarly, Bagdatli and Tastemel [26] and Erten et al. [27] demonstrated that aromatic and azo-containing structures can form strong complexes with Cu²⁺ and Ni²⁺ ions. These studies provide indirect evidence supporting the metal-binding tendency of phenolic and flavonoid structures.

The high TPC value (175.967 mg GAE/g) suggests the presence of numerous active phenolic binding sites in the biomass capable of interacting with metal ions. Likewise, elevated TFC (110.189 mg QE/g) is an important factor that may enhance biosorption efficiency owing to the metal-chelating capacity of flavonoids.

Biosorption is widely regarded as an eco-friendly and sustainable strategy for the removal of heavy metals from contaminated aquatic environments, as it relies on natural or modified biological materials and substantially reduces the need for harsh chemical reagents commonly employed in conventional remediation processes [33]. In addition to its environmental compatibility, biosorption offers clear economic advantages, since many biosorbents can be obtained from low-cost agricultural by-products or waste materials, making this approach particularly attractive for regions with limited financial resources [34, 35]. Notably, biosorbents have demonstrated high efficiency in reducing metal ion concentrations to trace levels, with numerous studies reporting strong adsorption capacities for toxic heavy metals such as Pb, Cd, and Cr, underscoring their suitability for water treatment applications [36, 37].

Within this context, several species of the genus *Salvia* have attracted attention for their potential roles in heavy metal biosorption and phytoremediation. Among them, S. *officinalis has* been shown to effectively accumulate toxic metals such as Pb and Cd, with UV–Vis spectroscopic studies confirming metal–phytochemical interactions and complex formation, thereby supporting its applicability in bioremediation systems [38]. Extracts of *Salvia* miltiorrhiza have also been reported to interact with Cu and Hg ions, presumably through hydroxyl and carboxyl functional groups, although the existing evidence remains largely indirect and warrants further targeted investigation [39]. Similarly, preliminary studies on *Salvia* virgata suggest its ability to sequester Cd and Pb through biomass–metal interactions, although comprehensive mechanistic insights remain limited [40]. In the case of *Salvia* sclarea, tolerance to elevated Zn levels and preferential accumulation of Zn in root tissues have been documented, indicating its potential suitability for remediation of Zn-contaminated soils under specific environmental conditions [41]. Although *Salvia* hispanica has been proposed as a potential biosorbent for Cd and Pb, current literature provides insufficient experimental validation, highlighting the need for more systematic studies to clarify its biosorption efficiency and underlying mechanisms [42].

**Fig. 2:**
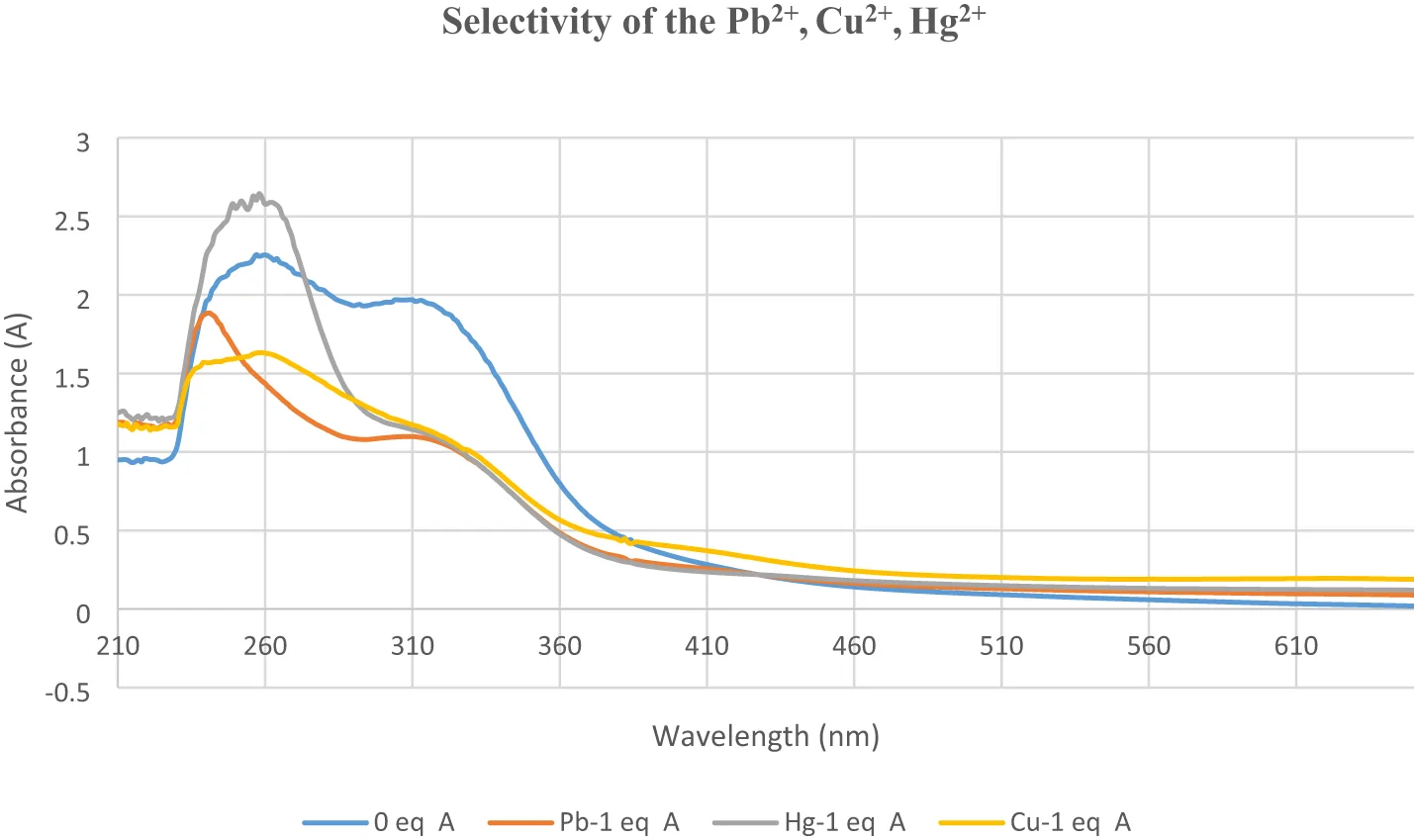
Selectivity of *S. officinalis* leaf extract toward Pb²⁺, Cu²⁺, and Hg²⁺ ions based on UV–Vis spectroscopic analysis

We determined the binding affinity of the prepared plant extract for the heavy metals lead (Pb), copper (Cu), and mercury (Hg) (Fig. 2). These results revealed that the phytochemicals in the extract interact significantly with these heavy metals, as indicated by affinity measurements. These results revealed that the phytochemicals in the extract interact significantly with these heavy metals, as indicated by affinity measurements as reported by Choi et al. [43]. The relative UV–Vis spectral response of the extract was evaluated by considering both the shift in the main absorption band and the absorbance changes at selected monitoring wavelengths. The free extract showed a main absorption maximum at 257 nm. After metal ion addition, this band shifted to 249 nm for Hg(II), 258 nm for Cu(II), and 242 nm for Pb(II), corresponding to Δλmax values of − 8, +1, and − 15 nm, respectively.

Job’s plot analysis for Pb(II) showed that the maximum absorbance change occurred at a metal mole fraction of approximately 0.67. Since the extract is a chemically complex matrix rather than a purified ligand, this result was interpreted as indicating an apparent metal-rich interaction tendency within the crude extract, rather than the formation of a discrete Pb–ligand complex. The apparent binding response of the Pb(II)–extract system was then evaluated using the extended Benesi–Hildebrand approach. The apparent binding response of the Pb(II)–extract system was subsequently evaluated using the extended Benesi–Hildebrand approach, based on the metal-rich interaction tendency suggested by Job’s plot analysis. A linear relationship was obtained in the plot of 1/ΔA versus 1/[Pb(II)]², supporting the applicability of this comparative spectroscopic model under the experimental conditions. The apparent binding parameter Kapp was calculated as 9.3 × 10⁻¹ M⁻². This value should not be interpreted as a true thermodynamic association constant, but rather as an apparent parameter reflecting the relative spectroscopic response of Pb(II) toward the crude extract matrix.

**Fig. 3:**
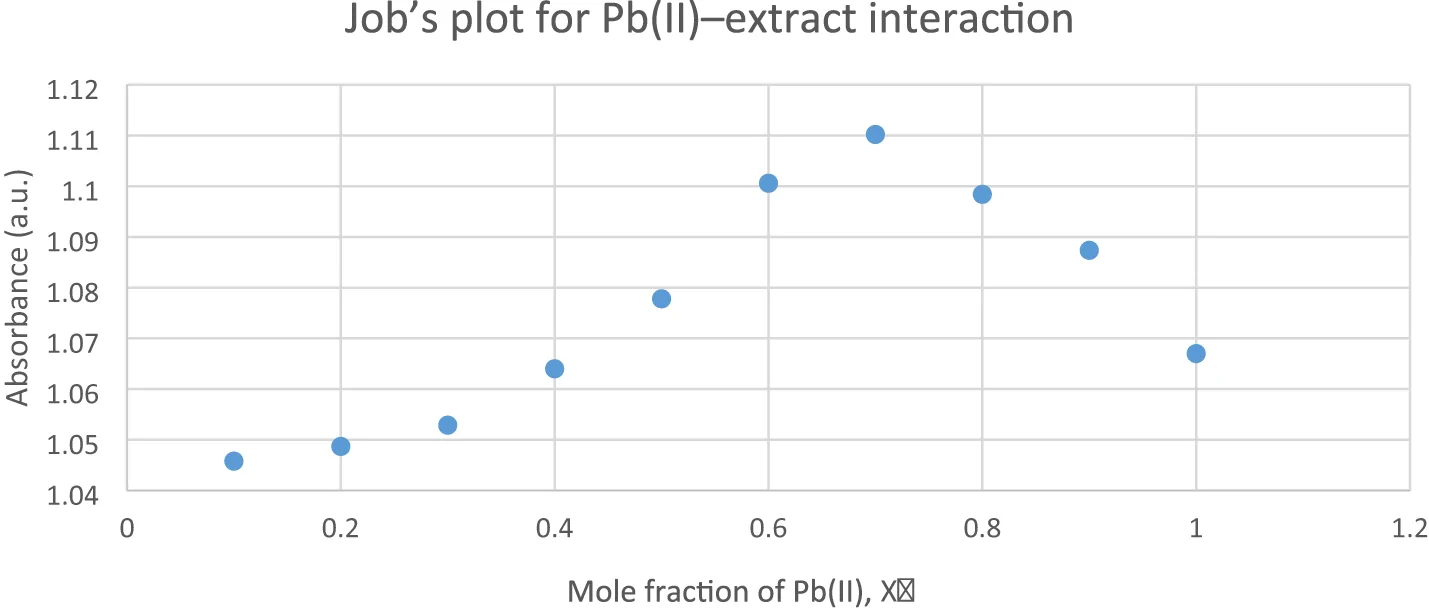
Job’s plot for Pb(II) binding to *S. officinalis* leaf extract based on UV–Vis spectroscopic data

**Fig. 4:**
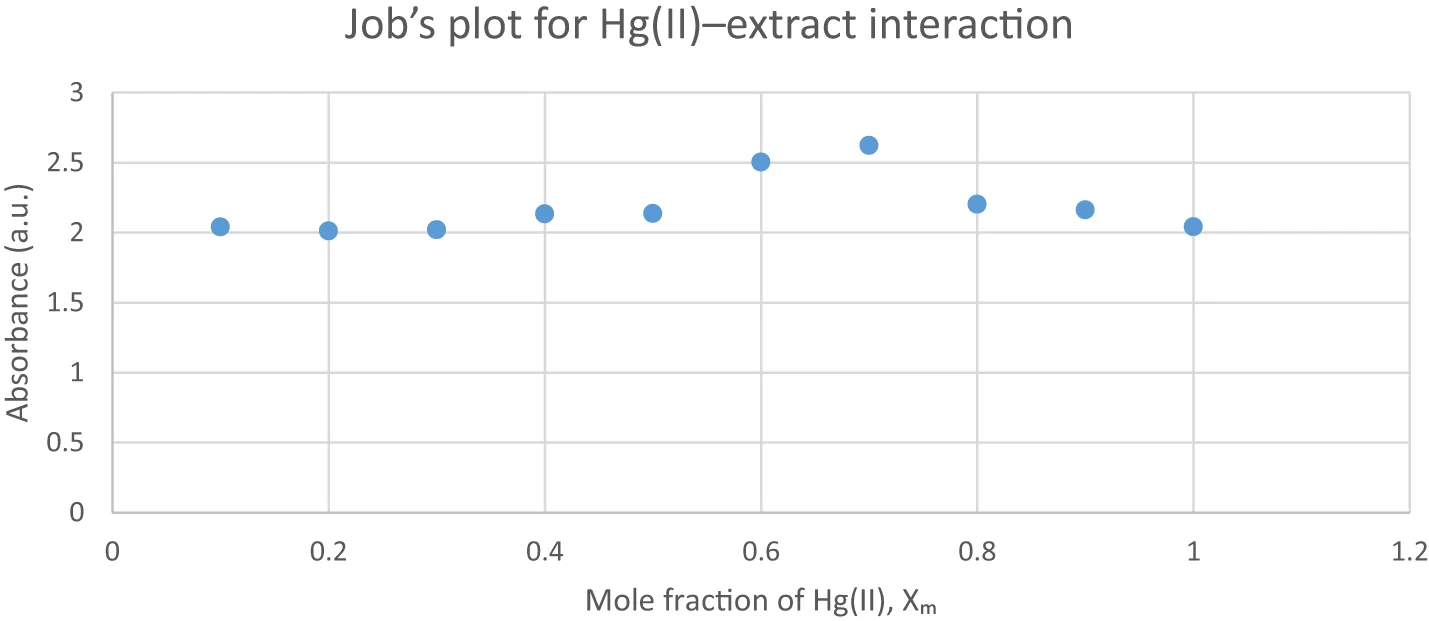
Job’s plot for Hg(II) binding to *S. officinalis* leaf extract based on UV–Vis spectroscopic data

A similar interaction trend was observed for Hg(II), for which Job’s plot indicated the maximum absorbance at a metal mole fraction of approximately 0.67. This result suggests an apparent metal-rich stoichiometric tendency within the crude extract matrix rather than the formation of a well-defined, discrete metal–ligand complex (Fig. 4). The extended Benesi–Hildebrand analysis yielded an apparent binding parameter of Kapp = 2.6 M⁻². This higher apparent value, compared with Pb(II), suggests a stronger spectroscopic interaction between Hg(II) and the crude extract matrix under the same experimental conditions. However, because the extract is not a purified ligand system, this value was interpreted only as an apparent comparative parameter rather than a true binding constant.

**Fig. 5:**
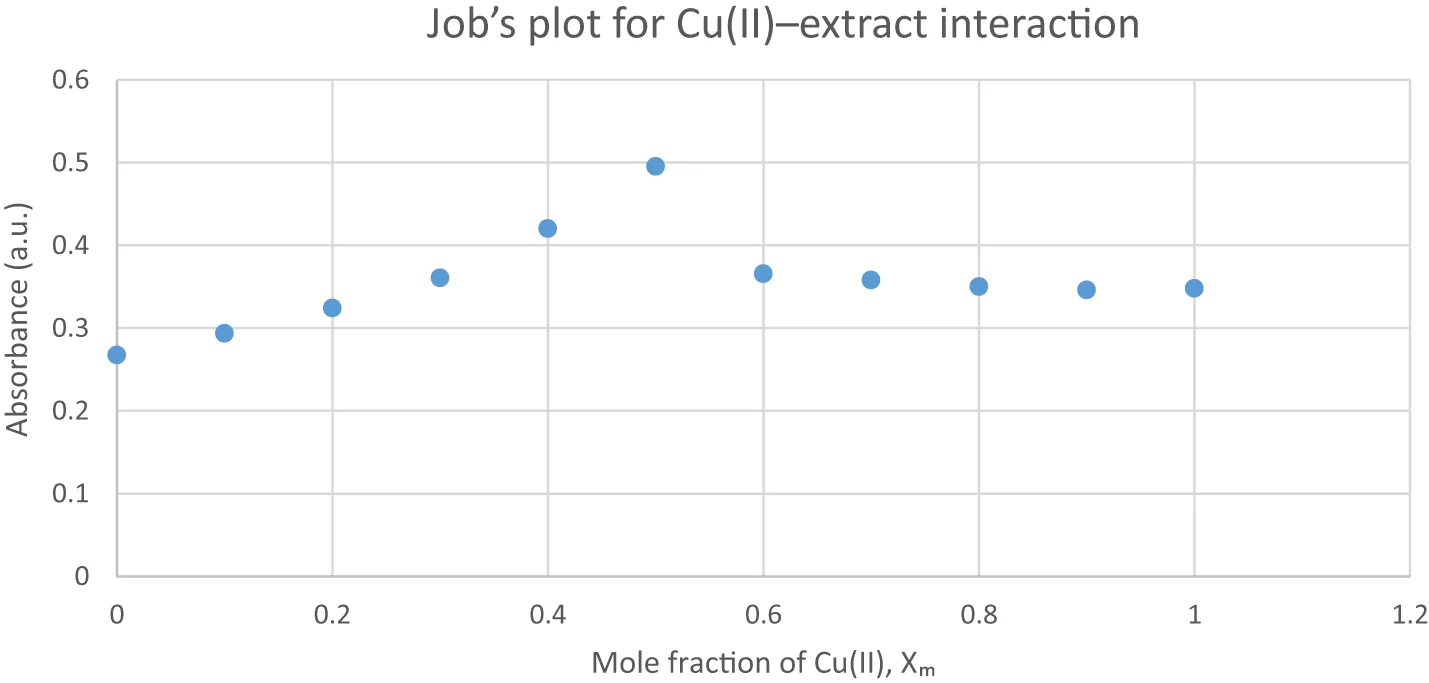
Job’s plot for Cu(II) binding to *S. officinalis* leaf extract based on UV–Vis spectroscopic data

In contrast, Job’s plot analysis for Cu²⁺ revealed a maximum absorbance at a metal mole fraction of 0.5, indicating a 1:1(M₁L) binding stoichiometry (Fig. 5). Consistent with this stoichiometry, the binding constant for the Cu–extract system was calculated using the Scatchard model, which is appropriate for ML-type interactions. The intrinsic binding constant was determined to be K = 2.25 M⁻¹, based on three independent measurements, with values expressed as the mean ± standard deviation. For Cu(II), Job’s plot analysis showed a maximum absorbance response at a metal mole fraction of approximately 0.50, suggesting a more balanced apparent metal–extract interaction profile. Consistent with this trend, the Cu(II)–extract system was evaluated using Scatchard analysis as a comparative approach. The obtained value was interpreted only as an apparent binding parameter, not as an intrinsic thermodynamic binding constant.

Overall, the comparative analysis of the three metal ions revealed distinct interaction behaviours toward *the S. officinalis* extract matrix. Job’s plot analysis suggested apparent metal-rich interaction patterns for Pb(II) and Hg(II), whereas Cu(II) exhibited a more balanced metal–extract interaction profile. The apparent binding parameters obtained from extended Benesi–Hildebrand analysis for Pb(II) and Hg(II), and the Scatchard-based apparent parameter for the Cu(II)–extract system, indicated that Hg(II) exhibited the strongest spectroscopic interaction, followed by Pb(II) and Cu(II). These values were used only for comparative purposes and should not be regarded as intrinsic thermodynamic constants, because the crude extract contains multiple phytochemical constituents and undefined binding sites (Table 3).

**Table 3:** Apparent stoichiometric pattern, Job plot maximum mole fractions, apparent binding parameters, and UV–Vis response characteristics for Pb(II), Hg(II), and Cu(II) interactions with *S. officinalis* leaf extract

Metal	Apparent stoichiometric pattern	Job Plot Xₘ(max)	Kapp Apparent binding parameter
Pb	Metal-rich apparent interaction (M₂L)	0.671	0.93 ± 0.1053
Hg	Metal-rich apparent interaction (M₂L)	0.678	1.02196 ± 0.0949
Cu	Balanced apparent interaction (M₁L)	0.5	1.02203 ± 0.1477

**Fig. 6:**
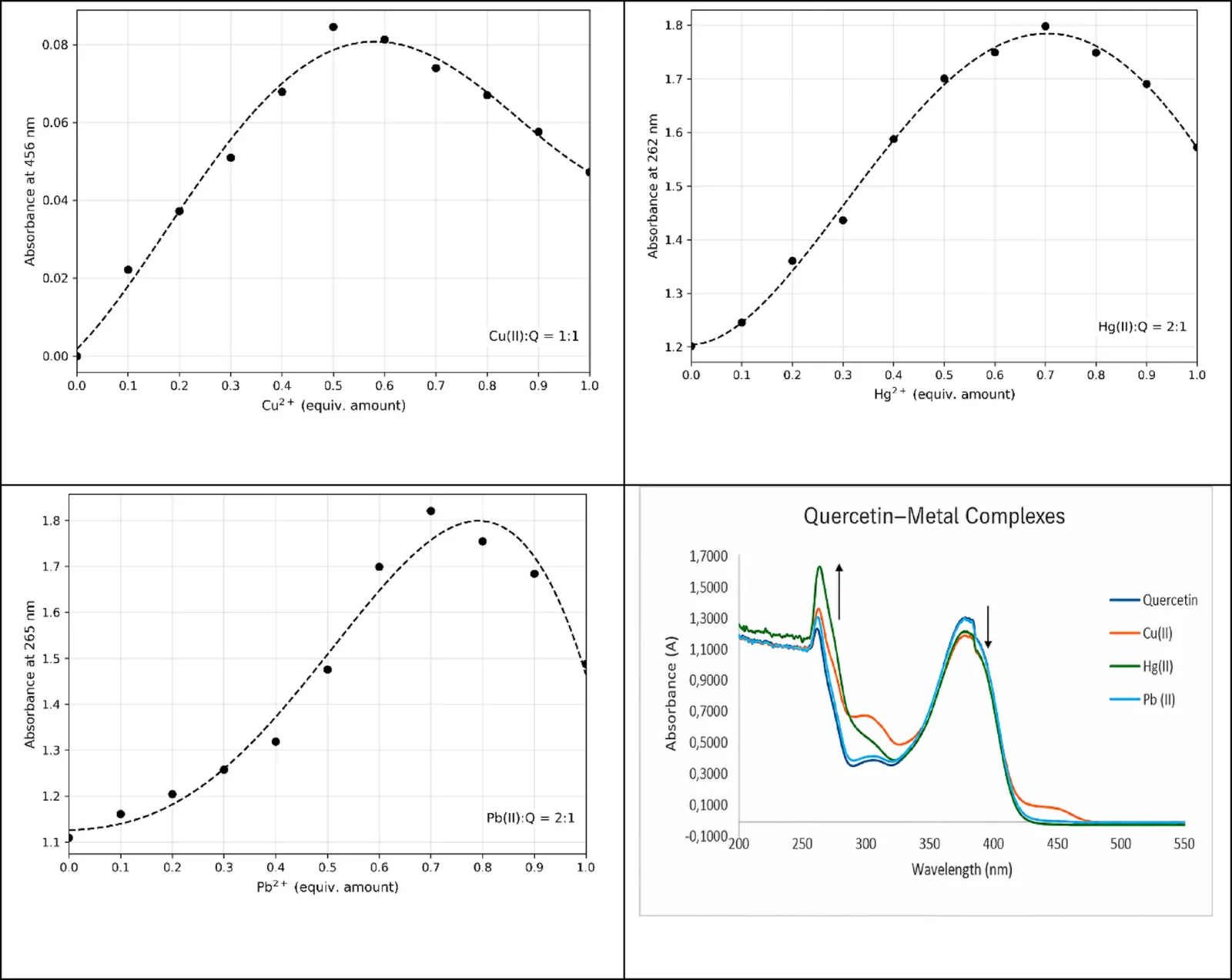
UV–Vis spectra and Job’s plot analyses of quercetin–metal complexes with Cu(II), Hg(II), and Pb(II)

To further support the interpretation of metal binding in the crude extract, quercetin was used as a structurally defined phytochemical control. Owing to its phenolic hydroxyl, carbonyl, and catechol moieties, which serve as donor sites, quercetin is expected to interact with metal ions through oxygen-based coordination. The addition of Cu(II), Pb(II), and Hg(II) induced clear changes in the UV–Vis spectrum of quercetin. In particular, an increase in the absorption band around 260–270 nm was observed upon metal addition, while the broader band at 370–390 nm showed a decrease or redistribution in absorbance (Fig. 6). In addition, the Cu(II)–quercetin complex exhibited a new shoulder at approximately 452 nm, further supporting the formation of a metal-induced electronic transition. These spectral changes suggest metal-induced alterations in the electronic environment of quercetin and support the formation of metal–quercetin complexes.

Job’s plot analysis indicated an apparent 1:1 stoichiometry for the Cu(II)–quercetin system, whereas Pb(II) and Hg(II) showed maxima at higher metal mole fractions, suggesting metal-rich stoichiometries close to M₂L under the applied conditions. These findings indicate that the UV–Vis approach is suitable for monitoring metal–phytochemical interactions. However, since the extract contains multiple phytochemical constituents with different donor groups, the quercetin data were evaluated as a structurally defined positive-control reference for comparison rather than as a direct model of the entire extract matrix.

The UV–Vis-derived apparent binding parameters, time-dependent absorbance responses, and ICP-OES-based retained metal contents describe different aspects of the metal–extract interaction. The apparent binding parameters reflect the relative spectroscopic interaction response in solution, whereas the time-dependent data describe the initial rate of spectral response, and the ICP-OES results indicate the amount of metal retained in the isolated extract-derived solid matrix. Accordingly, Hg(II) exhibited the strongest apparent spectroscopic binding response, Pb(II) and Hg(II) exhibited faster initial UV–Vis responses than Cu(II), and Pb(II) exhibited a higher retained metal content in the isolated solid system.

**Fig. 7:**
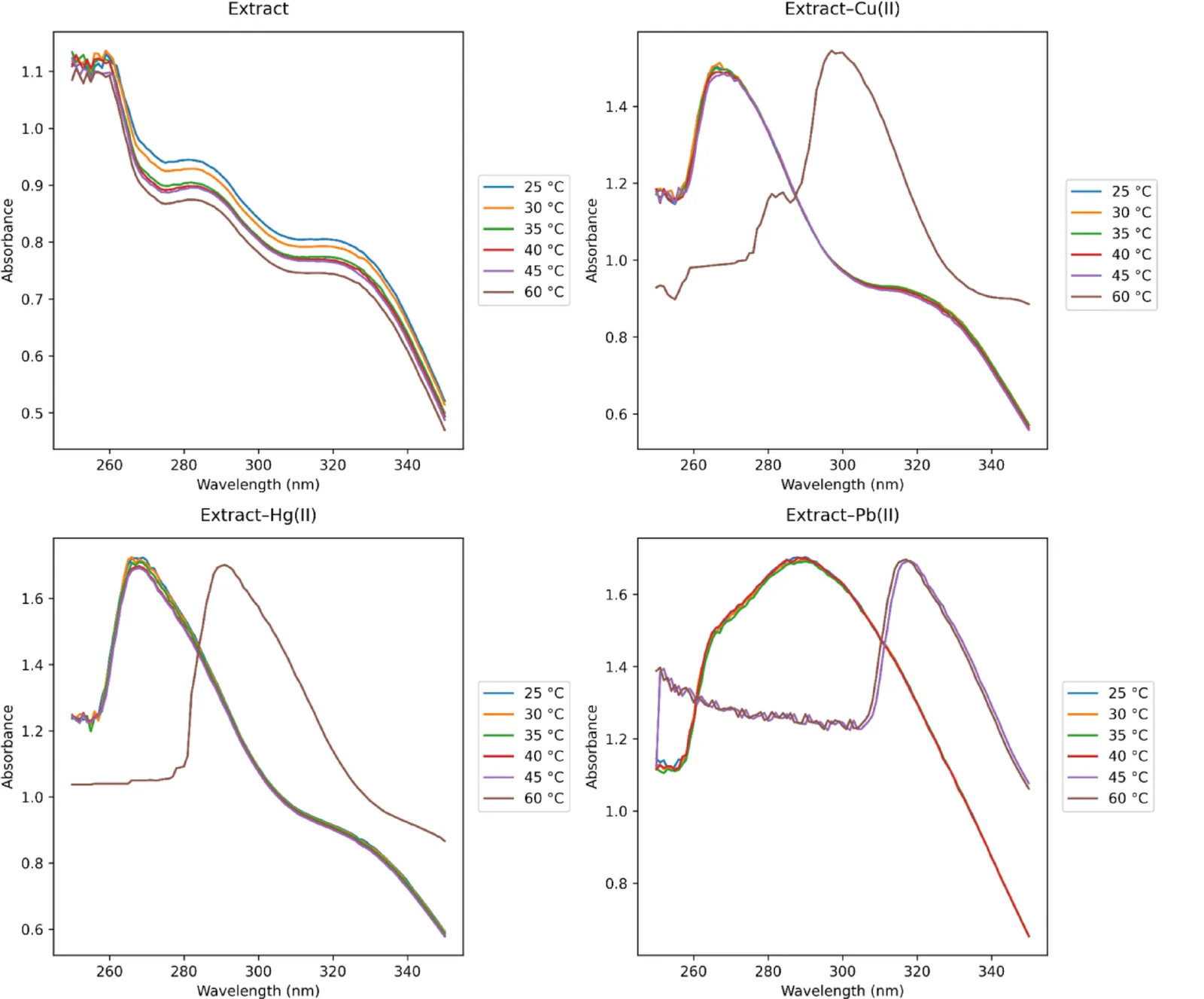
Temperature-dependent UV–Vis spectra of *S. officinalis* extract and its Cu(II), Hg(II), and Pb(II) metal–extract systems recorded between 25 and 60 °C. The observed bathochromic shifts were approximately 31 nm for Pb(II), 23 nm for Hg(II), and 24 nm for Cu(II). The spectral changes were interpreted as apparent temperature-dependent responses of the crude extract matrix

Temperature-dependent UV–Vis spectra *indicated* that both the free *S. officinalis* extract and the corresponding metal–extract systems underwent measurable spectral changes as temperature increased from 25 to 60 °C. The free extract displayed only a gradual decrease in absorbance with increasing temperature, suggesting that no major spectral disruption occurred under the applied conditions. In contrast, more pronounced changes were detected in the presence of Cu(II), Hg(II), and Pb(II), particularly above 45 °C. For the metal–plant extract systems, variations in absorbance intensity, band shape, and peak position were mainly observed in the 250–320 nm region, which is commonly associated with electronic transitions of aromatic and heteroatom-containing phytochemical constituents. These spectral variations indicate that temperature affects the apparent interaction environment between the metal ions and the crude extract matrix. The Pb(II), Hg(II), and Cu(II) systems showed bathochromic shifts of approximately 31, 23, and 24 nm, respectively, indicating temperature-dependent changes in the electronic environment of extract components involved in metal interactions. Nevertheless, these findings should be interpreted cautiously because the extract is a chemically complex matrix. The observed spectral changes cannot be attributed solely to stronger metal coordination or to a specific binding geometry. Although no visible turbidity was observed before UV–Vis measurements, partial aggregation or precipitation at higher temperatures cannot be completely excluded and may also contribute to the absorbance changes (Fig. 7). Therefore, the temperature-dependent UV–Vis data were interpreted as apparent spectral responses of the metal–extract systems under the applied experimental conditions, rather than as direct evidence of discrete thermally stabilized metal complexes.

The adsorption efficiency of plant extracts toward heavy metals, including Cu(II), Pb(II), and Hg(II), is influenced by pH, which governs both the ionization state of functional groups and proton competition at binding sites. Under acidic conditions, metal uptake is suppressed because H⁺ ions compete with metal cations for binding sites; conversely, uptake is favored at near-neutral to mildly alkaline pH (pH 6–10), where functional groups are more accessible for metal coordination. Excessively alkaline conditions may promote the precipitation of metal hydroxides, thereby reducing the effective adsorption. *S. officinalis* phytochemicals, rich in flavonoids, alkaloids, and terpenoids, exhibit characteristic UV–Vis responses that are sensitive to environmental pH [45]. Notably, alkaline conditions around pH 8 favour deprotonation of phenolic hydroxyl groups in flavonoids, leading to enhanced absorbance and bathochromic shifts in the 260–360 nm region [46, 47].

**Fig. 8:**
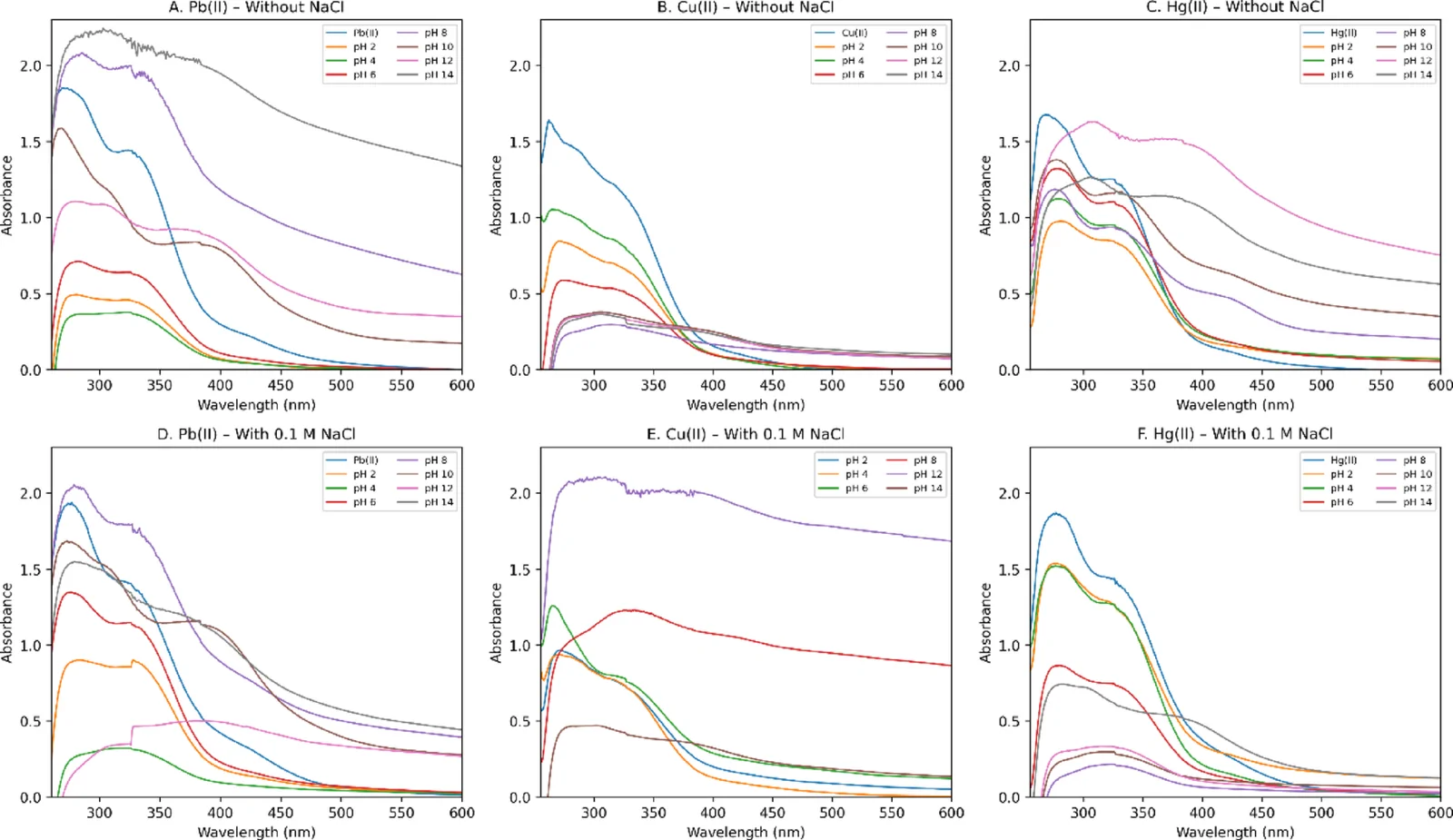
UV–Vis spectral responses of *S. officinalis* extract–metal systems under different pH and ionic strength conditions

In our study, the plant extract exhibited an intrinsic pH of 5.6 when dissolved in DMSO. UV–Vis measurements showed a gradual increase in absorbance at pH 2, 4, and 6, indicating enhanced electronic transitions under acidic to near-neutral conditions, consistent with pH-dependent protonation–deprotonation behaviour of phenolic and heteroatom-containing functional groups reported for plant-derived extracts [44, 45]. At pH 8, the extract exhibited both a bathochromic shift and a hypochromic effect, suggesting changes in the electronic environment of the chromophoric groups that are likely associated with deprotonation-induced structural rearrangements [46, 47]. When examining the pH-dependent behaviour of metal–extract systems, a pronounced hyperchromic effect was observed at pH 8 for all metal complexes, highlighting enhanced metal–ligand interactions under slightly alkaline conditions, consistent with previously reported pH-controlled biosorption and complexation mechanisms [48, 49]. Although a generally similar trend was observed across all metals, Cu(II) exhibited a distinct behaviour at pH 4, where the absorbance intensity exceeded that at pH 6 and displayed a hypsochromic shift, indicating a different coordination environment at this pH [50]. In contrast, both Pb(II) and Hg(II) exhibited a more pronounced bathochromic shift and a distinct hyperchromic response at pH 12 compared with acidic conditions, distinguishing their high-pH binding behaviour from that observed at lower pH values [51, 52]. (Fig. 8).

In the presence of 0.1 M NaCl, the pH-dependent UV–Vis response should not be interpreted solely as an ionic screening effect. The increased ionic strength may modify the activity coefficients of charged metal, ligand, and metal–ligand species, thereby shifting the apparent complexation equilibria and changing the predominant species at each pH. In addition, chloride ions may participate in metal coordination or compete with plant-derived donor groups, leading to mixed metal–chloride/ligand complexes with different spectral characteristics. Therefore, the different maximum responses observed under NaCl conditions, as the Pb(II) around pH 8, Cu(II) around pH 12, and Hg(II) around pH 2, are likely associated with metal-specific changes in speciation, ligand deprotonation, and possible chloride-stabilized coordination environments rather than a uniform pH-dependent binding trend [53–55].

**Fig. 9:**
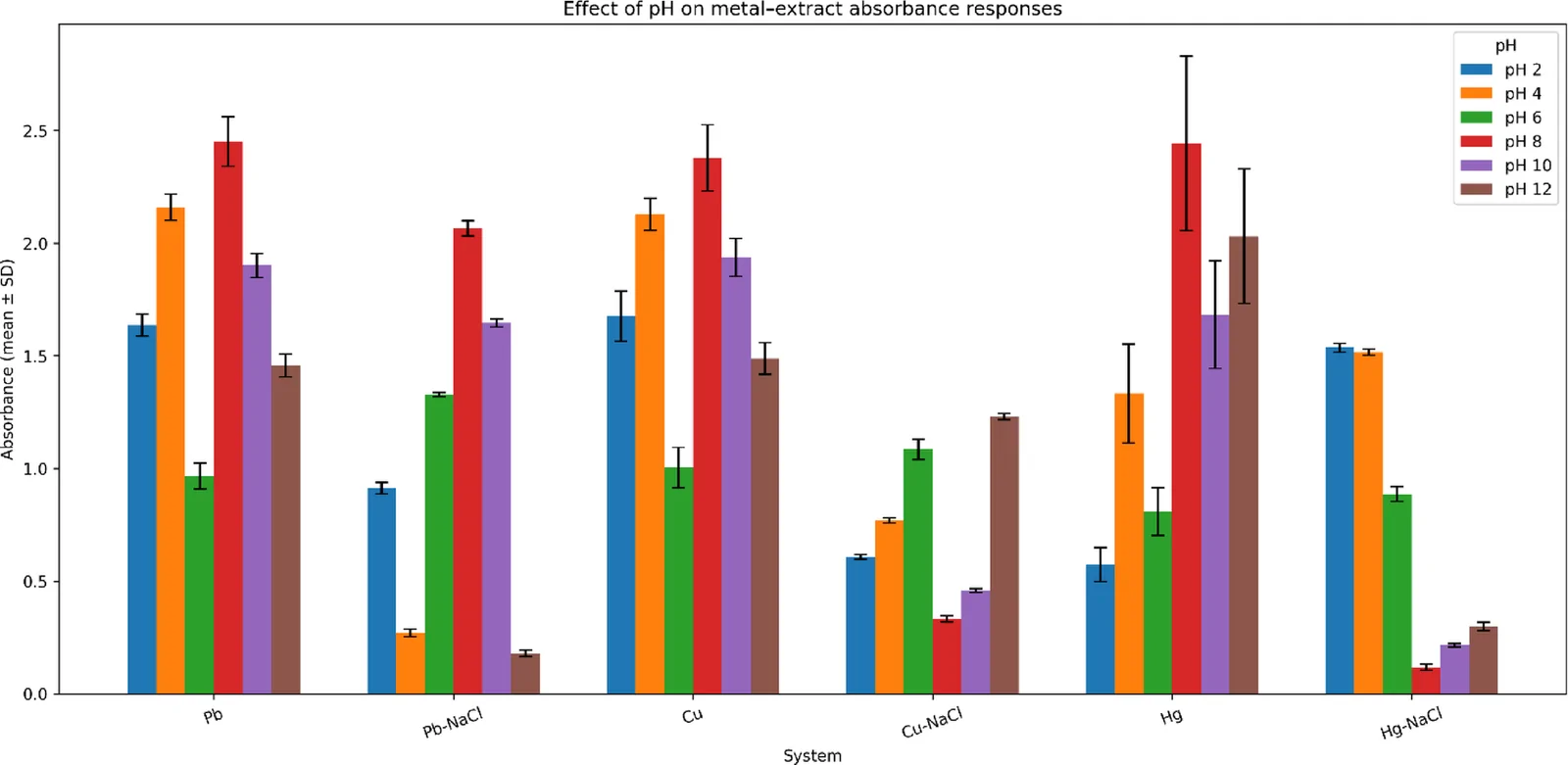
Effect of pH and ionic strength on the UV–Vis absorbance responses of *S. officinalis* extract complexes with Cu(II), Hg(II), and Pb(II)

One-way ANOVA demonstrated that pH significantly affected the UV–Vis absorbance values for all examined metal–extract studies, both in the absence and in the presence of 0.1 M NaCl (*p* < 0.05 in all cases). Tukey’s HSD post hoc analysis further indicated significant differences among several pH levels. However, the pH at which maximum absorbance occurred depended on ionic strength. In the NaCl-containing study, the highest mean absorbance was observed at pH 8 for Pb(II), pH 12 for Cu(II), and pH 2 for Hg(II), whereas, in the NaCl-free set, the maximum response for Pb(II), Cu(II), and Hg(II) occurred at pH 8 (Fig. 9). The presence of NaCl may influence the ionic strength of the medium, thereby modulating electrostatic interactions, shielding charged functional groups, and affecting metal ion availability.

**Fig. 10:**
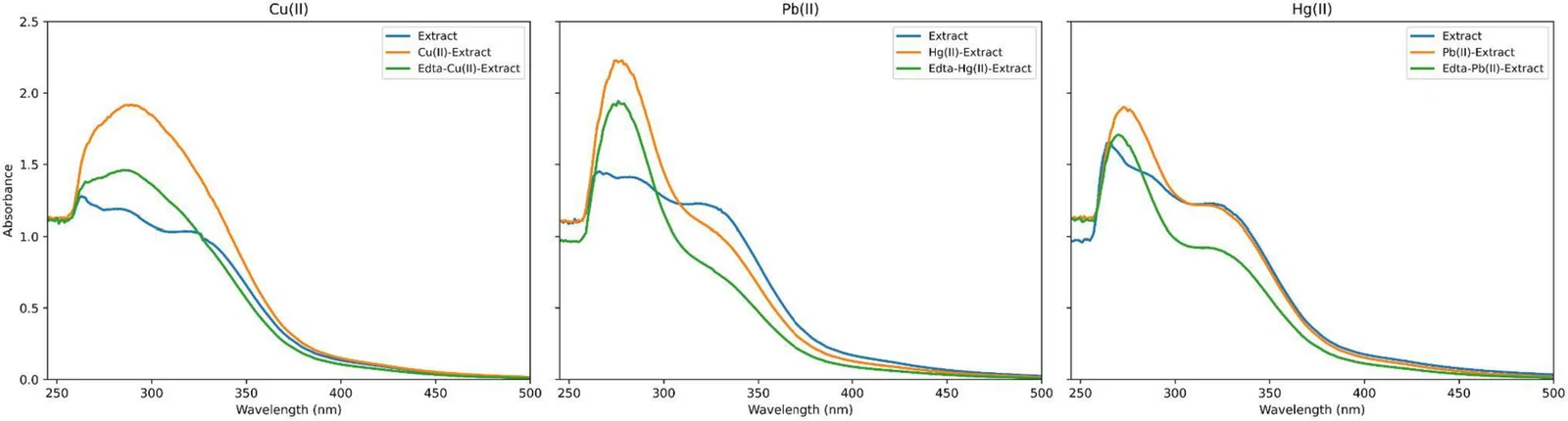
Reversibility of (Extract + Cu(II)/Hg(II)/Pb(II)) solution with the addition of EDTA

Plant extracts contain phytochemicals, such as polyphenols and flavonoids, that selectively bind heavy metals (e.g., Pb, Cu, Zn) via complexation and chelation [56]. The subsequent application of EDTA facilitates metal recovery by forming stable, soluble complexes that promote the desorption of ions from the plant-based matrix [57]. This synergistic effect ensures high reversibility, allowing for both efficient metal recycling and the regeneration of bio-adsorbents for repeated use [58]. The UV–Vis spectra revealed metal-dependent differences in EDTA-associated behaviour, with Hg(II)exhibiting the most pronounced absorbance changes, followed by Pb(II), and Cu(II) showing comparatively stable spectral profiles across the investigated wavelength range (Fig. 10).

Time-dependent UV–Vis measurements were performed at 35 °C and pH 5.6 to monitor the apparent rate of interaction between the metal ions and the *S. officinalis *extract matrix. For all metal–extract systems, absorbance increased rapidly during the initial stage and then approached a plateau after approximately 30 min, suggesting a rapid interaction followed by stabilization. The most pronounced initial response was observed for Pb(II) and Hg(II), whereas Cu(II) showed a more gradual increase. Based on the absorbance changes within the first 10 min, the initial apparent interaction response followed the order Pb(II) ≈ Hg(II) > Cu(II). However, after 60 min, the final absorbance response followed the order Hg(II) > Pb(II) > Cu(II), indicating a stronger overall spectral response for Hg(II) under the applied conditions. Because these measurements were based on UV–Vis absorbance changes rather than adsorption capacity, the data were not fitted to classical adsorption kinetic models, such as the pseudo-first-order and pseudo-second-order equations. Therefore, the results were interpreted as apparent time-dependent metal–extract interaction profiles rather than full adsorption kinetic parameters.

**Fig. 11:**
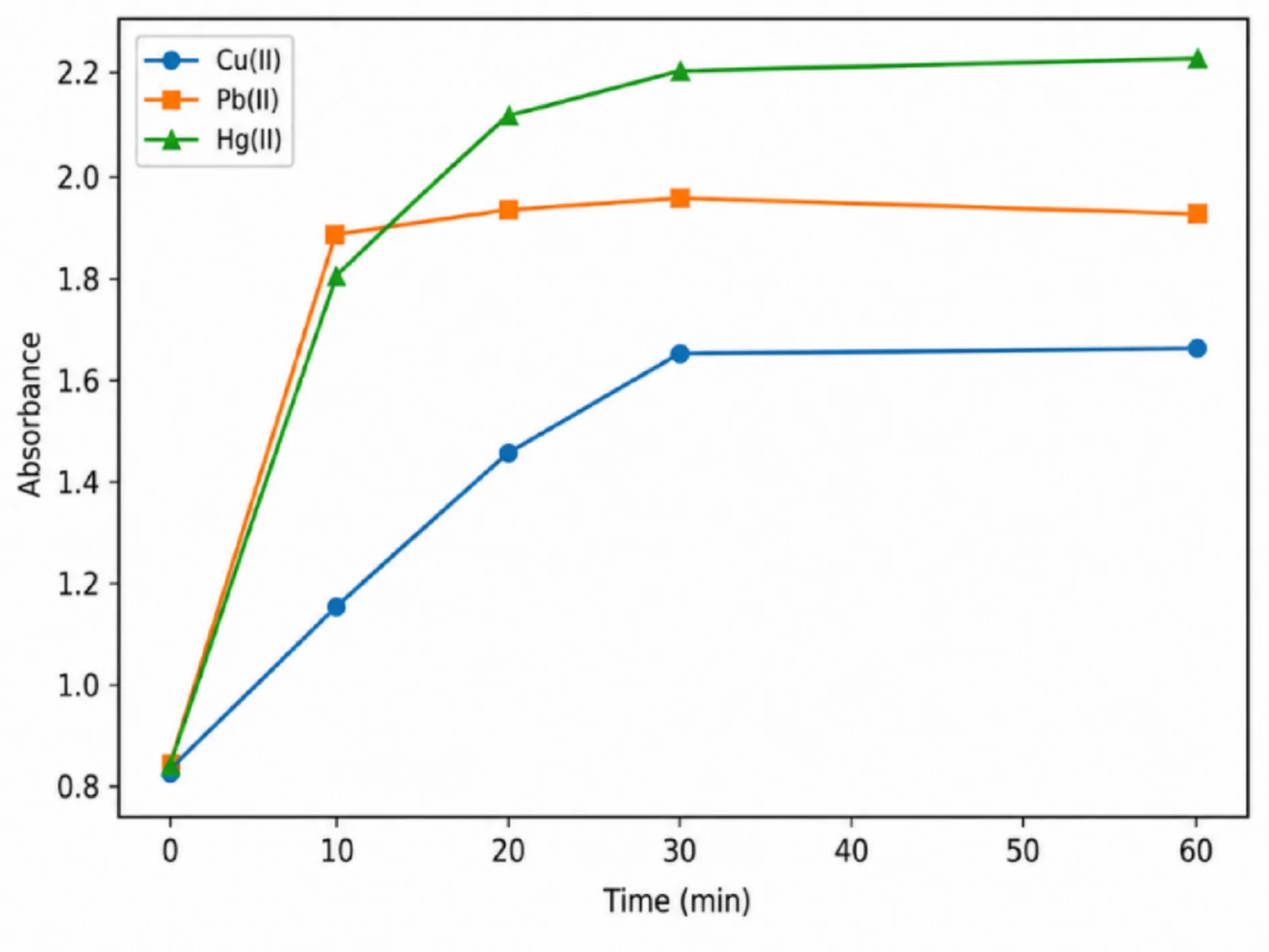
Time-dependent metal+extract absorbance value

To quantitatively support the metal–extract interaction behavior observed by UV–Vis spectroscopy, ICP-OES analysis was performed for the native *S. officinalis* extract and the isolated metal–extract systems. The ICP-OES instrument initially provided elemental concentrations in the digested solutions, reported as mg/L. To allow comparison between the native extract and the isolated metal–extract systems, these values were normalized to the initial sample mass and the final digestion volume and expressed as mg/kg in Table 3. The native extract contained low intrinsic levels of Cu and Pb, corresponding to 31.69 ± 0.78 mg/kg and 37.14 ± 0.26 mg/kg, respectively. After the metal–extract interaction and isolation of the solid extract-based systems, markedly higher metal contents were detected. The Cu content in the Cu(II)–extract system was calculated as 25,139.53 ± 4,023.26 mg/kg, whereas the Pb content in the Pb(II)–extract system reached 45,052.17 ± 2,739.13 mg/kg. These results support substantial incorporation of both metals into the extract-derived solid matrix, with a higher retained Pb content than that of Cu. However, this ICP-OES-based retained metal content should not be directly equated with the UV–Vis-derived apparent binding strength, since the two approaches reflect different experimental aspects of the metal–extract interaction.

**Table 4:** ICP-OES quantification of metal content in the *S. officinalis* extract and metal–extract complexes

Sample	Metal	Sample mass (mg)	Non-spiked digest concentration (mg/L)	Non-spiked content (mg/kg)	Spiked digest concentration (mg/L)	Spiked content (mg/kg)	Increase after spiking (mg/L)	Apparent recovery/response (%)	RSD* (%)
Plant extract	Cu	77	0.122 ± 0.003	31.69 ± 0.78	7.69 ± 2.00	1997.40 ± 519.48	+ 7.57	75.7	≈ 25.97
Plant extract	Pb	77	0.143 ± 0.001	37.14 ± 0.26	9.34 ± 0.03	2425.97 ± 7.79	+ 9.20	92.0	≈ 0.33
Cu–extract complex	Cu	86	108.1 ± 17.3	25139.53 ± 4023.26	143.1 ± 12.4	33279.07 ± 2883.72	+ 35.0	350.0	≈ 11–16
Pb–extract complex	Pb	92	207.24 ± 12.60	45052.17 ± 2739.13	254.58 ± 18.03	55343.48 ± 3919.57	+ 47.34	473.4	≈ 6–7

*Non-spiked samples are digested extract or metal–extract samples analyzed without the addition of an external standard. Spiked samples were prepared by external addition of a 10 ppm Cu(II) or Pb(II) standard to evaluate the analytical response and possible matrix effects. ICP-OES values were first obtained as digest solution concentrations(mg/L)and then converted to sample-normalized contents(mg/kg)using the initial sample mass and the final digestion volume of 20 mL

Standard addition was used to evaluate analytical response, recovery behaviour, and possible matrix effects during ICP-OES measurement. Since plant extracts contain complex organic and inorganic constituents, direct quantification may be affected by matrix-related signal suppression or enhancement. Therefore, known Cu(II) and Pb(II) standards were added to the digested samples to assess whether the analytical signal increased consistently under the same matrix conditions. The spiked digest concentrations increased from 0.122 ± 0.003 to 7.69 ± 2.00 mg/L for Cu and from 0.143 ± 0.001 to 9.34 ± 0.03 mg/L for Pb in the native extract. In the isolated metal–extract systems, the corresponding values increased from 108.1 ± 17.3 to 143.1 ± 12.4 mg/L for Cu and from 207.24 ± 12.60 to 254.58 ± 18.03 mg/L for Pb. These increases confirm that the ICP-OES method responded to standards added to the extract matrix. The relatively lower Cu response and higher variability may be associated with matrix effects, partial loss of more weakly retained Cu(II) species during washing, or comparatively weaker retention of Cu(II) by the crude extract matrix. Therefore, the standard addition results supported the reliability of metal quantification, allowing the ICP-OES data to be interpreted with caution.

The possible metal-binding mechanism of the *S. officinalis* extract can be attributed to the combined contributions of phenolic hydroxyl, carbonyl, carboxyl, amine, and C–O-containing functional groups present in the crude phytochemical matrix. These groups may participate in Pb(II), Cu(II), and Hg(II) binding through apparent coordination/chelation, electrostatic attraction, and possible ion-exchange-type interactions. Recent adsorption studies have emphasized that mechanistic interpretation should integrate spectroscopic evidence, elemental validation, pH-dependent behavior, and functional-group participation rather than relying only on removal efficiency or visual spectral changes [59, 60]. In the present study, the pH-dependent UV–Vis responses suggest that deprotonation of oxygen-containing groups under mildly alkaline conditions enhances metal interactions, while FTIR peak shifts and ICP-OES results support the involvement of these functional groups in metal incorporation into the extract-derived solid matrix. Therefore, the overall mechanism is best described as a matrix-level multi-site interaction process rather than as binding by a single purified ligand.

**Fig. 12:**
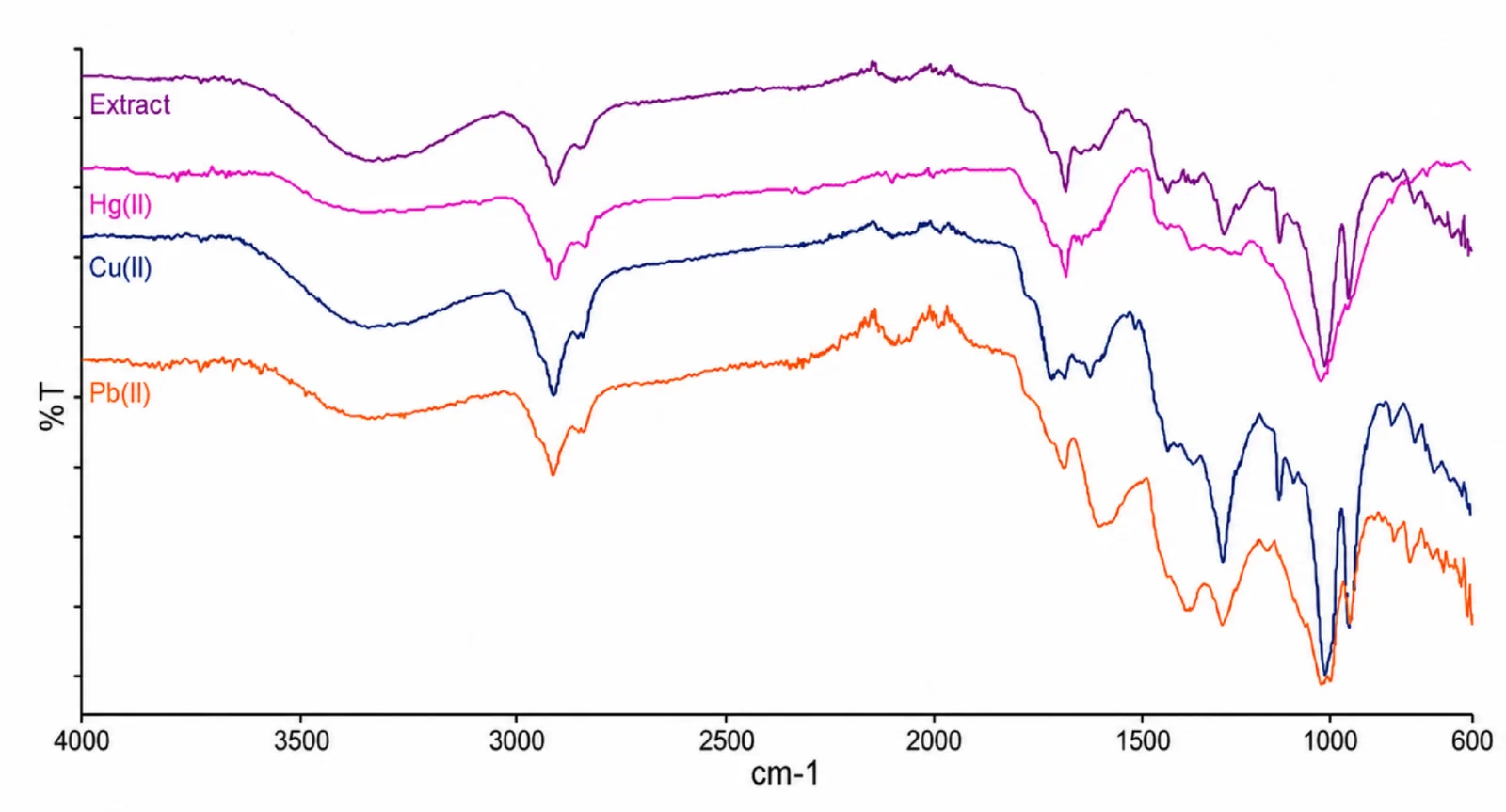
Comparative FTIR spectra of the *S. officinalis* extract and its Cu(II), Pb(II), and Hg(II) complexes

FTIR spectroscopy provided structural evidence for metal coordination in the extract-based complexes [61–64]. Comparison of the IR spectrum of the native Salvia *officinalis L. leaf extract* with the IR spectra of the Cu(II), Pb(II), and Hg(II)complexes revealed clear metal-dependent spectral changes, supporting the formation of metal–ligand interactions (Fig. 12).In the native extract, the broad band around 3374 cm⁻¹ was assigned to O–H and N–H stretching vibrations, while bands at 1790, 1723, and 1687 cm⁻¹ were associated with carbonyl-containing groups. The fingerprint region between 1200 and 900 cm⁻¹, particularly the bands at 1142, 1031, 999, and 952 cm⁻¹, was attributed mainly to C–O, C–O–C, C–OH, and C–COOH vibrations, including ring-coupled modes, of phenolic, alcoholic, etheric, and carboxylate-containing phytochemicals. The Cu(II) plant extract complex displayed the most pronounced decrease in %T in the 1200–900 cm⁻¹ region, indicating stronger absorption in this coordination-sensitive fingerprint zone. For instance, the average transmittance in the 1050–1020 cm⁻¹ region decreased from 61.44%T in the native extract to 51.60%T in the Cu(II)complex; the bands at 1289, 1142, 1031, 999, and 952 cm⁻¹ likewise showed lower %T values in the Cu(II) complex than in the native extract. This behavior indicates strong interactions of C–O-containing functional groups with Cu(II) and supports the involvement of phenolic, alcoholic, etheric, and carboxylate-related oxygen donors in Cu(II)binding. Importantly, this region should be interpreted as metal-perturbed C–O stretching and fingerprint vibrations, rather than as a direct M–O vibration. Literature data on *Salvia* extracts and plant polyphenols also place C–O/C–O–C and phenolic C–O vibrations within this region, while true M–O vibrations are more commonly supported by lower-frequency bands around 600–500 cm⁻¹ ([1.1], [3.1], [4.1]). The carbonyl region also changed after complexation. The Cu(II) complex showed stronger absorption near 1723 cm⁻¹ compared with the native extract, with %T decreasing from 87.45%T to 79.35%T; it also displayed a characteristic lower-wavenumber carbonyl-related band around 1632 cm⁻¹. This shift or reorganization is consistent with weakening or redistribution of the C = O bond upon metal coordination, supporting the participation of carbonyl and/or carboxylate oxygen atoms in Cu(II) binding. Similar carbonyl shifts have been reported for Cu(II)–flavonoid complexes, where C=O coordination causes a shift to lower wavenumber and is accompanied by low-frequency Cu–O vibration ([2.1]). For Pb(II) and Hg(II), the numerical data indicated a different pattern. In the 1050–1020 cm⁻¹ region, the mean %T values increased to 82.12%T for Pb(II) and 91.40%T for Hg(II), compared with 61.44%T for the native extract. Therefore, these complexes should not be described as showing a simple increase in absorption intensity in this region. Instead, the data support attenuation and redistribution of the native C–O fingerprint bands, accompanied by the appearance of metal-specific features such as the Pb-associated 999 cm⁻¹ band and the Hg-associated 1031 cm⁻¹ band. These changes suggest that Pb(II) and Hg(II) coordinate with oxygenated phytochemical sites but induce different ligand environments, possibly due to differences in ionic radii, binding preferences, coordination geometries, and post-complexation purification procedures.The broad O–H/N–H region also supports metal-dependent coordination. At 3374 cm⁻¹, the native extract showed 87.27%T, whereas Hg(II) and Pb(II) exhibited higher values, 96.59%T and 94.53%T, respectively, indicating attenuation of the native hydroxyl/amine-related vibration. This suggests strong involvement or masking of hydroxyl and possibly amine-containing groups after Pb(II) and Hg(II) binding. In contrast, the Cu(II) complex showed a %T value at this position similar to that of the native extract but exhibited more pronounced changes in the C=O and C–O regions, suggesting that Cu(II) coordination is more clearly reflected in carbonyl/carboxylate and phenolic C–O perturbations.

**Table 5:** FTIR assignments and coordination-related spectral changes of *Salvia officinalis* L. metal complexes

Functional group / interaction region	*Salvia officinalis* L. leaf extract	Cu(II) complex	Pb(II) complex	Hg(II) complex
O–H / N–H stretching	3374 cm⁻¹: 88.90%T. Broad O–H stretching with possible N–H overlap.	3374 cm⁻¹: 86.84%T; shifted region at 3332 cm⁻¹: 86.74%T. Slightly stronger/shifted band suggests altered hydrogen bonding and O/N-donor involvement.	3374 cm⁻¹: 93.62%T. Attenuation indicates strong perturbation or masking of O–H/N–H groups after Pb binding.	3374 cm⁻¹: 96.36%T. Strong attenuation suggests pronounced involvement or suppression of O–H/N–H vibrations after Hg complexation.
Aliphatic C–H stretching	2961: 90.10%T; 2927: 86.19%T; 2860: 90.49%T. Assigned to –CH₂/–CH₃ stretching.	2950: 80.69%T; 2927: 76.16%T. Stronger C–H-region absorption indicates reorganization of aliphatic phytochemical components, not direct Cu–C bonding.	2950: 92.07%T; 2927: 90.38%T. Weaker/attenuated C–H contribution compared with Cu, suggesting a different retained organic environment.	2950: 95.14%T; 2927: 94.17%T. Marked attenuation indicates lower contribution or masking of aliphatic vibrations in the Hg complex.
C = O stretching / carbonyl–carboxyl region	1790: 95.62%T; 1723: 89.96%T; 1687: 85.43%T. Carbonyl groups from ester, acid, lactone, or conjugated C = O structures.	1726: 79.38%T; 1723: 78.83%T; 1632: 79.70%T. Stronger absorption and lower-wavenumber C = O-related band support Cu coordination through carbonyl/carboxyl oxygen donors.	1726: 92.21%T; 1687: 90.75%T; 1632: 89.16%T. Carbonyl envelope is attenuated/reorganized, supporting Pb-induced modification rather than simple intensification.	1726: 95.52%T; 1687: 94.33%T; 1632: 95.77%T. Strong attenuation of native carbonyl bands suggests marked redistribution or masking of C = O containing groups after Hg binding.
COO⁻ / phenolate-associated bending or stretching	1435 cm⁻¹: 85.32%T. CH bending with possible symmetric COO⁻ and phenolate/ring contribution.	1434 cm⁻¹: 69.43%T. Stronger absorption supports alteration of carboxylate/phenolate-associated vibrations after Cu coordination.	1434 cm⁻¹: 84.34%T. Similar to native, but slightly altered; suggests moderate Pb effect on COO⁻/phenolate-related vibrations.	1434 cm⁻¹: 95.74%T. Attenuation suggests this native vibration is suppressed or replaced by a different Hg-specific fingerprint.
Phenolic C–O / C–OH / C–O–C stretching	1289: 79.87%T; 1142: 78.73%T. C–O-rich region of phenolic, alcoholic, etheric, and carboxylate-related groups.	1289: 56.86%T; 1142: 66.01%T. Strong decrease in %T indicates clear perturbation of C–O-containing donor groups, supporting Cu–O coordination through phenolic/enolic/alcoholic oxygen sites.	1289: 81.53%T; 1142: 85.37%T. Attenuation compared with native suggests Pb-induced redistribution of C–O bands rather than stronger absorption.	1289: 94.99%T; 1142: 94.08%T. Strong attenuation suggests that native C–O bands are masked, weakened, or reorganized in the Hg complex.
900–1200 cm⁻¹ metal-sensitive C–O fingerprint region	1050: 73.12%T; 1031: 68.11%T; 1020: 64.94%T; 999: 67.76%T; 952: 72.22%T. Assigned to C–O, C–O–C, C–OH, C–COOH, and ring-coupled vibrations.	1050: 60.99%T; 1031: 51.67%T; 1020: 47.05%T; 999: 47.08%T; 952: 52.01%T. The strongest absorption in this region supports strong Cu-induced perturbation of C–O/C–COOH groups and possible C–O–Cu linkage formation.	1050: 80.02%T; 1031: 78.35%T; 1020: 77.87%T; 999: 77.97%T; 952: 81.92%T. Pb shows a distinct fingerprint profile, especially around 999 cm⁻¹, consistent with Pb-specific changes in C–O/carboxylic/ring-coupled vibrations.	1050: 91.21%T; 1031: 90.62%T; 1020: 90.89%T; 999: 91.49%T; 952: 93.18%T. The native C–O fingerprint region is strongly attenuated; Hg-associated 1031 cm⁻¹ should be interpreted as metal-perturbed C–O vibration, not pure M–O stretching.
Pb-specific C–O / C–COOH / ring-coupled vibration	952–999 cm⁻¹ region: 72.22–67.76%T. Native C–COOH/ring-coupled fingerprint.	999 cm⁻¹: 47.08%T. Cu also strongly affects this region, but not as a Pb-specific marker.	999 cm⁻¹: 77.97%T. Pb-specific spectral feature indicates modification of carboxylic/C–O/ring-coupled vibrations, supporting Pb binding through oxygen donor groups.	999 cm⁻¹: 91.49%T. Strong attenuation; Hg does not show the same Pb-type fingerprint behavior at this position.
Aromatic C–H out-of-plane / substituted aromatic ring vibrations	827: 86.03%T; 777: 84.73%T; 733: 82.77%T. Aromatic phenolic fingerprint region.	827: 72.73%T; 777: 74.20%T; 733: 70.24%T. Stronger bands indicate retention of aromatic phenolic constituents and altered aromatic ligand environment after Cu binding.	827: 86.65%T; 777: 84.59%T; 733: 85.69%T. Similar or slightly attenuated profile; suggests weaker aromatic fingerprint modification than Cu.	825: 96.87%T; 777: 97.38%T; 733: 97.62%T. Strong attenuation, but Hg-specific bands indicate a distinct aromatic ligand environment after purification/complexation.
Low-frequency M–O / metal–ligand region	571: 72.90%T; 525: 81.03%T. Low-frequency region relevant to M–O/M–ligand support.	571: 63.01%T; 525: 64.72%T. Stronger low-frequency absorption supports Cu–O/metal–ligand vibration formation.	571: 80.15%T; 525: 71.38%T. Modification in the low-frequency region supports Pb–ligand interaction, especially near 525 cm⁻¹.	No clear Excel value at 571/525 cm⁻¹ in the provided Hg column. Therefore, Hg–O evidence should be discussed mainly through O–H, C = O, and C–O fingerprint changes, not low-frequency bands.
Hg-specific overtone / combination region	1995: 99.93%T; 1981: 100.02%T; 1975: 100.33%T. Very weak/near-baseline region in native extract.	1995: 99.37%T; 1981: 99.35%T; 1975: 100.28%T. No major diagnostic Cu assignment.	1995: 98.78%T; 1981: 99.22%T; 1975: 99.66%T. Weak changes; not sufficient for donor assignment.	1995: 97.91%T; 1981: 97.96%T; 1975: 97.83%T. Hg-specific weak bands may reflect a distinct vibrational environment but should be interpreted cautiously and not used alone to assign a new donor group.

Finally, the lower-frequency region, around 525–571 cm⁻¹, provides the most direct evidence for metal–oxygen or metal–ligand bond formation. The Cu(II) complex showed lower %T than the native extract at 571 cm⁻¹ and 525 cm⁻¹, indicating stronger contributions from low-frequency metal–ligand vibrations. Taken together, the FTIR data indicate that the *Salvia* extract coordinates Cu(II), Pb(II), and Hg(II) mainly through phenolic/alcoholic O–H, carbonyl/carboxylate C = O/COO⁻, and C–O containing functional groups, while the distinct spectral behavior in the 900–1200 cm⁻¹ region demonstrates metal-specific coordination and ligand reorganization after complex formation and purification.

The stronger response of *S. officinalis* extract toward Cu(II), Pb(II), and Hg(II) is likely related to the compatibility of these ions with the donor groups present in the crude extract matrix. Oxygen- and nitrogen-containing phytochemical derivatives, such as phenolic –OH, carbonyl, carboxyl, amine, and C–O sites, can support metal-dependent interactions, while the larger size and greater polarizability of Pb(II) and Hg(II) may favor multi-site binding compared with the more specific coordination behavior of Cu(II).The different apparent coordination behaviours of Pb(II), Hg(II), and Cu(II) can be attributed to differences in their ionic characteristics and coordination structures. Pb(II) and Hg(II), being larger and more polarizable, may interact with multiple donor groups within the crude extract matrix, resulting in apparently more metal-rich stoichiometric trends and more pronounced FTIR changes. In contrast, Cu(II) showed a more balanced apparent coordination profile, which may be associated with its smaller ionic radius and coordination geometry. These differences support the view that metal binding by *S. officinalis* extract occurs through a matrix-level multi-site mechanism rather than a single coordination atom or group. Although flavonoids are important contributors to metal coordination, other phytochemical classes in the crude extract matrix, including phenolic acids, tannin-like compounds, alkaloid-type constituents, terpenoids and oxygen- or nitrogen-containing metabolites, may also provide active donor groups for Pb(II), Cu(II), and Hg(II)binding.

## Conclusion

This study provides a mechanistic evaluation of the complexation behavior of *Salvia officinalis* leaf extract toward Pb(II), Cu(II), and Hg(II). The spectroscopic results showed clear metal-dependent differences in the extract response. Job’s plot analysis suggested an apparent M₂L-type complexation for Pb(II) and Hg(II), whereas Cu(II) showed an apparent 1:1 coordination mode under the selected experimental conditions. ICP-OES analysis further supported the metal-binding capacity of the extract and indicated a higher accumulation of Pb than of Cu. The complexation behavior was influenced by pH, ionic strength, and temperature. The stronger response observed under neutral to mildly alkaline conditions may be associated with the deprotonation of hydroxyl, carbonyl, phenolic, and other heteroatom-containing functional groups. Temperature-dependent UV–Vis measurements also showed more pronounced spectral changes above 45 °C, suggesting that temperature can affect the metal–extract interaction. In addition, EDTA-induced desorption indicated that the binding process was at least partly reversible, supporting a coordination-based interaction rather than irreversible metal retention.

To strengthen the interpretation of metal binding, quercetin was included as a structurally defined phytochemical positive control. The UV–Vis spectral changes and Job’s plot results obtained for quercetin–metal systems confirmed that the applied spectroscopic approach was suitable for monitoring metal–phenolic interactions and provided a useful reference for interpreting the more complex behaviour of the crude extract matrix. Overall, the UV–Vis and FTIR findings suggest that hydroxyl, carbonyl, amine-related, and phenolic donor groups in the phytochemical matrix contribute to metal coordination. The present findings provide a mechanistic evaluation of the complexation behaviour between* S. officinalis* leaf extract and metal ions. These results may provide a useful basis for future studies on environmental metal removal.

## Novelty statement

This study provides a comparative, mechanistic evaluation of interactions of Pb(II), Cu(II), and Hg(II) with *Salvia officinalis *L. leaf extract in a solution-phase system. By integrating UV–Vis spectroscopic stoichiometry, apparent binding analysis, ICP-OES quantification, and FTIR functional-group assignments, this study clarifies metal-dependent interaction patterns within a chemically complex plant-extract matrix. The findings reveal distinct, apparent binding behaviors, with Pb(II) and Hg(II) showing M₂L-type interaction patterns and Cu(II) exhibiting an apparent 1:1 binding mode under controlled pH and temperature. EDTA-assisted reversibility further supports the regenerative potential of the extract-based metal-binding system, and the results provide a basis for future studies of plant-derived materials for sustainable heavy-metal remediation.

## Data Availability

All data used and generated in this study are included in the article.

## References

[1] Mallongi A, et al. Environmental sustainability and human health risks of toxic metals. J Environ Health. 2022;84:12–8. 10.1016/j.jenvman.2022.10112.

[2] Staszak M, Regel-Rosocka M. Plant tissues and biological materials as effective biosorbents. Biomed Res Int. 2024;2024:8841235. 10.1155/2024/8841235.

[3] Rubio-Vargas DA, Morais TP, Randi MAF, Filipak Neto F, Martins CC, Oliveira AP et al. Pollutant bioaccumulation in sentinel fish chronically exposed in Iguaçu river reservoirs (Southern Brazil) and human health risk of fish consumption. Environ Pollut. 2024;357:124660. 10.1016/j.envpol.2024.124660. 38036225

[4] Vialkova E, Korshikova E, Fugaeva A. Phytosorbents in wastewater treatment technologies. Rev Water. 2024;16(18):2626. 10.3390/w16182626.

[5] Sheraz M, Liotta LF, Akram M, Wang R. Next-generation Z-scheme and S-scheme heterojunction photocatalysts for microplastics mineralization. Coord Chem Rev. 2026;557:217735. 10.1016/j.ccr.2025.217735.

[6] Asiminicesei DM, Fertu DI, Gavrilescu M. Impact of heavy metal pollution in the environment on the metabolic profile of medicinal plants and their therapeutic potential. Plants. 2024;13(6):913. 10.3390/plants13060913. PMC10976221 38592933

[7] Kumar S, Anmol A, Wani AW, Bakshi M, Dhiman Z. Recent advancements in deciphering the therapeutic properties of plant secondary metabolites: phenolics, terpenes, and alkaloids. Plant Physiol Biochem. 2024;211:108674. 10.1016/j.plaphy.2024.108674. 38705044

[8] Abouzeid S, Lewerenz L, Yahyazadeh M, Radwan A, Hijazin T, Kleinwächter M, Selmar D. Favorable impacts of drought stress on the quality of medicinal plants: improvement of composition and content of their natural products. In: Aftab T, editor. Environmental Challenges and Medicinal Plants: Sustainable Production Solutions under Adverse Conditions. Cham: Springer; 2022. pp. 105–31. 10.1007/978-3-030-92050-0_4.

[9] Moshari-Nasirkandi A, Iaccarino N, Romano F, Graziani G, Alirezalu A, Alipour H, Amato J. Chemometrics-based analysis of the phytochemical profile and antioxidant activity of Salvia species from Iran. Sci Rep. 2024;14(1):17317. 10.1038/s41598-024-68421-8. PMC11283568 39068233

[10] EFSA Panel on Additives and Products or Substances used in Animal Feed (FEEDAP), Villa RE, Azimonti G, Bonos E, Christensen H, Durjava M, Dusemund B, Gehring R, Glandorf B, Kouba M, López-Alonso M, Marcon F, Nebbia C, Pechová A, Prieto-Maradona M, Röhe I, Theodoridou K, Bastos ML, Benfenati E, Brantom P, Chesson A, Schlatter J, Westendorf J, Manini P. Safety and efficacy of a feed additive consisting of a tincture derived from the leaves of Salvia officinalis L. (sage tincture) for use in all animal species (FEFANA asbl). EFSA J. 2025;23(7):e9545. 10.2903/j.efsa.2025.9545 PMC12308210 40741369

[11] Aldaddou WA, Aljohani ASM, Ahmed IA, Al-Wabel NA, El-Ashmawy IM. *officinalis* L. methanolic extract reduces lead and nicotine-induced sperm quality degeneration in male rats. Chem Biodivers. 2023;20(7):e202300115. 10.1002/cbdv.202300115. 37236909

[12] Aydin D, Yalçin E, Çavuşoğlu K. Metal chelating and anti-radical activity of *Salvia officinalis* in the ameliorative effects against uranium toxicity. Sci Rep. 2022;12(1):15845. 10.1038/s41598-022-20115-9 PMC9508101 36151120

[13] Pirsaheb M, Gholami T, Seifi H, Dawi EA, Said EA, Hamoody AHM, Altimari US, Salavati-Niasari M. Green synthesis of nanomaterials by using plant extracts as reducing and capping agents. Environ Sci Pollut Res. 2024;31(17):24768–87. 10.1007/s11356-024-32983-x. 38523214

[14] Ahmed A, Usman M, Yu B, Ding X, Peng Q, Shen Y, Cong H. Efficient photocatalytic degradation of toxic Alizarin Yellow R dye from industrial wastewater using biosynthesized Fe nanoparticle and study of factors affecting the degradation rate. J Photochem Photobiol B Biol. 2020;202:111682. 10.1016/j.jphotobiol.2019.111682. 31731077

[15] Usman M, Ahmed A, Hu ZF, Shen YQ, Yu B, Cong HL. Synthesis, characterization and photocatalytic activity of iron nanoparticles from *Ficus carica* peels via biological method. Ferroelectrics. 2019;548(1):89–96. 10.1080/00150193.2019.1587423.

[16] Kongdee A, Bechtold T. Influence of ligand type and solution pH on heavy metal ion complexation in cellulosic fibre: model calculations and experimental results. Cellulose. 2009;16(1):53–63. 10.1007/s10570-008-9244-9.

[17] Zhang W, Huang H, Tan F, Wang H, Qiu R. Influence of EDTA washing on the species and mobility of heavy metals residual in soils. J Hazard Mater. 2010;173(1–3):369–76. 10.1016/j.jhazmat.2009.08.068. 19748734

[18] Aityoub A, Abouelfida A, Benyaich A, Elmeray M, Idouhli R, Koumya Y, Khadiri M. Hierarchization of physicochemical factors during simultaneous removal of lead and copper from the Marrakech municipal discharge soils. J Chem. 2020;2020:1–10. 10.1155/2020/4167897.

[19] Johnson M, et al. Stability constants of divalent and trivalent metal-EDTA complexes. Coord Chem Rev. 2002;228(2):185–92. 10.1016/S0010-8545(02)00015-X.

[20] Park YS, et al. Determination of total flavonoid content using quercetin reference standard. J Agric Food Chem. 2008;56(4):1120–7. 10.1021/jf072456b.

[21] Folin O, Ciocalteu V. On tyrosine and tryptophane determinations in proteins. J Biol Chem. 1927;73(2):627–50.

[22] Slinkard K, Singleton VL. Total phenol analysis: Automation and comparison with manual methods. Am J Enol Vitic. 1997;28(1):49–55. 10.5344/ajev.1977.28.1.49.

[23] Pedersen CJ. Ionic complexes of macrocyclic polyethers. Fed Proc. 1968;27:1305–9. 5725220

[24] Valverde Castilla GA, Mira McWilliams JM, González-Pérez B. One-Layer vs. Two-Layer SOM in the Context of Outlier Identification: A Simulation Study. Appl Sci. 2021;11(14):6241. 10.3390/app11146241.

[25] Sytar O, Brestic M, Rai M, Zivcak M. Spectroscopic approach for analysis of total phenolic content in plant extracts. Environ Exp Bot. 2014;107:147–53. 10.1016/j.envexpbot.2014.05.007.

[26] Beck MT, Nagypál I. Chemistry of Complex Equilibria. Ellis Horwood Series in Inorganic Chemistry. New York: Halsted Press / John Wiley & Sons; 1990.

[27] Erten G, Karcı F, Demirçalı A, Söyleyici S. 1H-pyrazole-azomethine based novel diazo derivative chemosensor for the detection of Ni2+. J Mol Struct. 2020;1206:127713. 10.1016/j.molstruc.2020.127713.

[28] Scatchard G. The attractions of proteins for small molecules and ions. Ann N Y Acad Sci. 1949;51(4):660–72. 10.1111/j.1749-6632.1949.tb27297.x.

[29] Kfoury M, Landy D, Fourmentin S. Characterization of cyclodextrin/volatile inclusion complexes: a review. Molecules. 2018;23(5):1204. 10.3390/molecules23051204. PMC6100373 29772824

[30] Foo KY, Hameed BH. Insights into the modeling of adsorption isotherm systems. Chem Eng J. 2010;156:2–10. 10.1016/j.cej.2009.09.013.

[31] Wojdyło A, Oszmiański J, Czemerys R. Antioxidant activity and phenolic compounds in 32 selected herbs. Food Chem. 2007;105(3):940–9. 10.1016/j.foodchem.2007.04.038.

[32] Foo KY, Hameed BH. Insights into the modeling of adsorption isotherm systems. Chem Eng J. 2010;156(1):2–10. 10.1016/j.cej.2009.09.013.

[33] Ahluwalia SS, Goyal D. Microbial and plant derived biomass for removal of heavy metals from wastewater. Bioresour Technol. 2007;98(12):2243–57. 10.1016/j.biortech.2005.12.006. 16427277

[34] Demirbas A. Heavy metal adsorption onto agro-based waste materials: A review. J Hazard Mater. 2008;157(2–3):220–9. 10.1016/j.jhazmat.2008.01.024. 18291580

[35] Babel S, Kurniawan TA. Low-cost adsorbents for heavy metals uptake from contaminated water: a review. J Hazard Mater. 2003;97(1–3):219–43. 10.1016/S0304-3894(02)00263-7. 12573840

[36] Sud D, Mahajan G, Kaur MP. Agricultural waste material as potential adsorbent for sequestering heavy metal ions from aqueous solutions: a review. Bioresour Technol. 2008;99(14):6017–27. 10.1016/j.biortech.2007.11.064. 18280151

[37] Ma D, Weir MH, Hull NM. Fluence-based QMRA model for bacterial photorepair and regrowth in drinking water after decentralized UV disinfection. Water Res. 2023;231:119612. 10.1016/j.watres.2023.119612. 36706469

[38] Arkas M, Tsiourvas D. Organic/inorganic hybrid nanospheres based on hyperbranched poly(ethylene imine) encapsulated into silica for the sorption of toxic metal ions and polycyclic aromatic hydrocarbons from water. J Hazard Mater. 2009;170(1):35–42. 10.1016/j.jhazmat.2009.05.031. 19493619

[39] Deng S, Ting YP. Characterization of PEI-modified biomass and biosorption of Cu(II), Pb(II) and Ni(II). Water Res. 2005;39(10):2167–77. 10.1016/j.watres.2005.03.001. 15927227

[40] Ismail T, Lee HK, Lee H, Kim Y, Kim E, Lee JY, et al. Early life exposure to perfluorooctanesulfonate (PFOS) impacts vital biological processes in Xenopus laevis: integrated morphometric and transcriptomic analyses. Ecotoxicol Environ Saf. 2024;269:115820. 10.1016/j.ecoenv.2023.115820. 38103469

[41] Saraswat S, Rai JPN. Phytoextraction potential of six plant species grown in multimetal contaminated soil. Chem Ecol. 2009;25(1):1–11.

[42] Oluyemisi AB, Nkechi EE, Oluwasegun AA, Simeon JJ, Basirat OA, Oluwadamilola AH, Osonuga IO. Investigating the total phenolic contents, in-vitro antioxidant activities and elemental contents of Annona muricata, Bryophyllum pinnatum and Syzygium aromaticum in South-West Nigeria. Afr J Pure Appl Sci. 2024;5(1):45–54. 10.33886/ajpas.v5i1.493.

[43] Choi S, Choi KH, Won J, Kim H. Impact of multi-heavy metal exposure on renal damage indicators in Korea: An analysis using Bayesian kernel machine regression. Medicine. 2023;102(41):e35001. 10.1097/md.0000000000035001. PMC10578771 37832107

[44] Zhang L, Liu Y, Wang Y. Deprotonation mechanism of methyl gallate: UV spectroscopic and computational studies. Int J Mol Sci. 2018;19(10):3111. 10.3390/ijms19103111. PMC6213618 30314293

[45] Agatonovic-Kustrin S, Gegechkori V, Kustrin E, Morton DW. The Effect of Lactic Acid Fermentation on Extraction of Phenolics and Flavonoids from Sage Leaves. Appl Sci. 2022;12(19):9959. 10.3390/app12199959.

[46] Maleš Z, Bojić M, Radić Brkanac S, Juričić J. Quantitative analysis of polyphenols and antioxidant activity of Fraxinus ornus L. leaves. Acta Pharm. 2023;73(2):315–26. 10.2478/acph-2023-0018.

[47] Candra KA, Rahayu ML, Saputra KAD, Dewantara IPS. Kandungan fitokimia dan uji aktivitas antibakteri kombinasi ekstrak daun lemon balm (*Melissa officinalis*) dan daun sage (*Salvia officinalis*) terhadap bakteri Streptococcus pyogenes penyebab tonsilitis. Intisari Sains Medis. 2024;15(3):1384–9. 10.15562/ism.v15i3.2228.

[48] Vijayaraghavan K, Yun YS. Bacterial biosorption and a discussion on alternative biosorbents for heavy metal wastewater treatment. Biotechnol Adv. 2008;26(3):266–91. 10.1016/j.biotechadv.2008.01.002. 18353595

[49] Álvarez-Diduk R, Ramírez-Silva MT, Galano A, Merkoçi A. Deprotonation mechanism and acidity constants in aqueous solution of flavonols: a combined experimental and theoretical study. J Phys Chem B. 2013;117(41):12347–59. 10.1021/jp4051046. 24063416

[50] Sheng PX, Ting YP, Chen JP. Biosorption of heavy metal ions (Pb, Cu, and Cd) from aqueous solutions by the marine alga Sargassum sp. in single- and multiple-metal systems. Ind Eng Chem Res. 2007;46(8):2438–44. 10.1021/ie061071n.

[51] Liu X, Tang J, Wang Y, Zhang L. A novel turn-on fluorescent sensor based on carbon dots for the highly sensitive and selective detection of levofloxacin. Spectrochim Acta Part A Mol Biomol Spectrosc. 2022;268:120684. 10.1016/j.saa.2021.120684.

[52] Elsayed A, Moussa Z, Alrdahe SS, Alharbi MM, Ghoniem AA, El-Khateeb AY, Saber WI. Optimization of heavy metals biosorption via artificial neural network: A case study of cobalt (II) sorption by Pseudomonas alcaliphila NEWG-2. Front Microbiol. 2022;13:893603. 10.3389/fmicb.2022.893603. PMC9194897 35711743

[53] Sipos P. Application of the Specific Ion Interaction Theory (SIT) for the ionic products of aqueous electrolyte solutions of very high concentrations. J Mol Liq. 2008;143(1):13–6.

[54] Crea F, De Stefano C, Irto A, Lando G. Acid-base properties of dimethyltin(IV) and complexing ability towards chloride in aqueous solution at different ionic strengths and temperatures. Mar Chem. 2020;225:103842. 10.1016/j.marchem.2020.103842.

[55] Azad M, Dreybrodt W, Scholz D. Kinetic controls on the carbon isotope composition of speleothems: a laboratory approach. Geochim Cosmochim Acta. 2023;348:115–28. 10.1016/j.gca.2023.02.011.

[56] Cherrak SA, Mokhtari-Soulimane N, Berroukeche F, Bensenane B, Cherbonnel A, Merzouk H, Elhabiri M. In vitro antioxidant versus metal ion chelating properties of flavonoids: a structure–activity investigation. PLoS ONE. 2016;11(10):e0165575. 10.1371/journal.pone.0165575. PMC5082868 27788249

[57] Sheng PX, Ting YP, Chen JP. Biosorption of heavy metal ions (Pb, Cu, and Cd) from aqueous solutions by the marine alga Sargassum sp. in single-and multiple-metal systems. Ind Eng Chem Res. 2007;46(8):2438–44.

[58] Takayanagi T, Motomizu S. Synergistic effects and reversibility in bio-adsorbent regeneration. Anal Sci. 2002;18(9):985–92. 10.2116/analsci.18.985.

[59] Bulin C, Guo T, Bao J. PMIDA modified Zr-BDC-NH₂ complex for efficient capture of cerium from water: Mechanistic insights from statistical physics and spectroscopy. Chem Eng J. 2025;522:167883. 10.1016/j.cej.2025.167883.

[60] Bulin C, Ma Y, Guo T. Adsorption behaviour and mechanism of Gd(III) over EDTA functionalized MIL-101-graphene oxide. Water Res. 2025;283:123841. 10.1016/j.watres.2025.123841. 40393334

[61] Perron NR, Brumaghim JL. A review of the antioxidant mechanisms of polyphenol compounds related to iron binding. Cell Biochem Biophys. 2009;53(2):75–100. 10.1007/s12013-009-9043-x. 19184542

[62] Celiz G, Suarez SA, Arias A, Molina J, Brondino CD, Doctorovich F. Synthesis, structural elucidation and antiradical activity of a copper (II) naringenin complex. Biometals. 2019;32(4):595–610. 10.1007/s10534-019-00192-5. 30911923

[63] Salman S, et al. Weakening of carbonyl bonds upon metal coordination: An IR study. J Mol Struct. 2019;1185:210–8. 10.1016/j.molstruc.2019.02.074.

[64] Espina A, Sanchez-Cortes S, Jurašeková Z. Vibrational study (Raman, SERS, and IR) of plant gallnut polyphenols related to the fabrication of iron gall inks. Molecules. 2022;27(1):279. 10.3390/molecules27010279. PMC8746386 35011511

